# Pre- and post-diagnosis signalling lipid profiles in heart failure with preserved ejection fraction: a prospective cohort study

**DOI:** 10.1016/j.ebiom.2026.106235

**Published:** 2026-03-27

**Authors:** Lu Zhang, Tamas Szili-Torok, Lieke Lamont, Alida Kindt, Amy Harms, Stephan J.L. Bakker, Ron Gansevoort, Martin H. de Borst, Thomas Hankemeier

**Affiliations:** a Metabolomics and Analytics Centre, Leiden Academic Centre for Drug Research (LACDR), Leiden University, Leiden, the Netherlands; b Department of Internal Medicine, Division of Nephrology, University Medical Center Groningen, University of Groningen, Groningen, the Netherlands

**Keywords:** HFpEF, Signalling lipids, Metabolomics, Oxylipins, Lysophospholipids, Eicosanoids

## Abstract

**Background:**

Heart failure with preserved ejection fraction (HFpEF) is a prevalent syndrome with limited diagnostic and therapeutic options. Dyslipidaemia is a common comorbidity, and signalling lipids, bioactive molecules regulating key pathophysiological processes, are implicated in HFpEF, though their relationships remain unclear.

**Methods:**

Plasma samples from the PREVEND cohort were analysed, including 172 control samples, 125 PreDx-HFpEF samples (before diagnosis), and 30 PostDx-HFpEF samples (after diagnosis, paired with PreDx-HFpEF). A total of 261 signalling lipids were profiled via LC-MS/MS. Additionally, sums and ratios of specific signalling lipid targets were calculated. Cox proportional hazards and logistic regression models were performed to identify relationships between signalling lipids and HFpEF progression.

**Findings:**

PreDx-HFpEF individuals showed elevated non-esterified oxylipins (derived from DHA, AA, EPA) and specific lysophospholipids, compared with controls, and these signalling lipids emerged as potential predictors of incident HFpEF. In contrast, the PostDx-HFpEF subgroup demonstrated an attenuation of non-esterified oxylipins and lysophospholipids compared with control and PreDx-HFpEF samples, although findings are exploratory given the limited sample size. Among 103 detected non-esterified oxylipins, 44 showed significant associations with HFpEF-related outcomes. Together with 18 additional signalling lipids, this yielded 62 significant signalling lipids, 68% of which were independent of high-density lipoprotein (HDL).

**Interpretation:**

Selected signalling lipids provide predictive information prior to HFpEF diagnosis, whereas post-diagnostic lipid alterations reflect disease-associated metabolic changes and should be interpreted cautiously. These findings highlight the potential relevance of signalling lipids in HFpEF.

**Funding:**

This study was funded by Netherlands Heart Foundation, Chinese Scholarship Council, and Dutch Research Council.

**Research in context**

**Evidence before this study**

Existing evidence indicates that HFpEF is increasingly recognised as a disorder driven by systemic inflammation, metabolic dysregulation, endothelial dysfunction, and myocardial fibrosis. Although conventional lipids such as cholesterol and triglycerides have been investigated in HFpEF, prior studies have not comprehensively profiled bioactive signalling lipids or examined how these lipids evolve from preclinical to established HFpEF. No available study has evaluated paired pre-diagnosis and post-diagnosis lipidomic samples in the same individuals. Evidence regarding oxylipins and lysophospholipids is fragmented, limited to small cross-sectional cohorts, and lacks temporal characterisation. Thus, substantial gaps remain in understanding how systemic signalling lipids change during HFpEF progression and which lipid species may serve as early predictors.

**Added value of this study**

This study provides a longitudinal lipidomic characterisation of HFpEF progression using paired samples from a large community-based cohort. By profiling 261 signalling lipids, including free oxylipins, lysophospholipids, free fatty acids, bile acids, oxysterols, and steroids, we identify distinct metabolic phases before and after HFpEF diagnosis.

We show that PreDx-HFpEF individuals demonstrate elevated DHA-, AA-, and EPA-derived oxylipins and selected lysophospholipids, several of which predict HFpEF incidence up to 8 years prior to diagnosis with AUC values around 0.8. After diagnosis, these signalling lipids exhibit a striking reversal, becoming markedly reduced in PostDx-HFpEF samples. Importantly, 68% of the significant lipid alterations were independent of HDL, despite HDL itself showing reversed trends after diagnosis.

We also identify two consistently altered lipid markers across all comparisons, 10-HDoHE/DHA and LPE 18:0, which represent robust indicators of HFpEF progression. Collectively, these findings provide insights into dynamic lipid signalling shifts during HFpEF development and establish a mechanistic framework for the involvement of oxylipins and lysophospholipids in cardiovascular inflammation, endothelial dysfunction, and myocardial remodelling.

**Implications of all the available evidence**

The integrated evidence suggests that HFpEF is characterised by staged and reversible disturbances in signalling lipid metabolism, reflecting distinct biological transitions from early metabolic dysregulation to established disease. The identification of predictive oxylipins and lysophospholipids highlights their potential utility for early detection, risk stratification, and monitoring of HFpEF progression, particularly in individuals with metabolic risk factors.

The observed lipid reversal after HFpEF diagnosis indicates a shift in systemic inflammatory and oxidative stress pathways that may have implications for disease staging and treatment monitoring. Moreover, because most lipid alterations occurred independently of HDL, therapies targeting specific bioactive lipid pathways may provide benefits beyond traditional lipid-lowering strategies.

These findings underscore the need for future mechanistic studies to explore how signalling lipid networks contribute to HFpEF pathogenesis and to evaluate whether modifying these pathways could enable earlier diagnosis or enhance therapeutic precision.

## Introduction

Heart failure with preserved ejection fraction (HFpEF) is a highly prevalent clinical syndrome, affecting approximately 32 million people worldwide.^1^^,^^2^ Its incidence continues to rise, largely driven by population ageing and the growing burden of comorbidities such as hypertension, obesity, diabetes, and metabolic syndrome.^2^ Clinically, HFpEF is defined as heart failure symptoms in the presence of a normal or near-normal left ventricular ejection fraction (LVEF ≥ 50%). Pathophysiologically, it is primarily characterised by left ventricular diastolic dysfunction, typically accompanied by myocardial stiffness and impaired ventricular filling.^3^ Patients with HFpEF experience significant morbidity and mortality, with recurrent hospitalisations and poor quality of life, resulting in a substantial healthcare burden.^1^, ^2^, ^3^, ^4^

Despite its high prevalence, HFpEF remains a diagnostic and therapeutic challenge. The syndrome exhibits heterogeneous phenotypes and is often complicated by overlapping comorbidities, rendering diagnosis difficult and potentially requiring advanced imaging or invasive haemodynamic assessments.^5^ Current therapeutic options offer limited benefit compared to those for heart failure with reduced ejection fraction (HFrEF), defined by LVEF < 50%. This therapeutic gap highlights an urgent need for deeper insights into its diagnosis and pathophysiology.^6^

Among the diverse mechanisms underlying HF, the role of lipids has gained increasing attention in recent years. Signalling lipids, bioactive lipid molecules, act as dynamic messengers in inter- and intracellular communication, regulating processes such as inflammation, cell growth, apoptosis, and vascular tone.^7^ Notably, key pathophysiological features of HFpEF, including systemic inflammation, endothelial dysfunction, and myocardial fibrosis, are tightly regulated by bioactive lipid signalling,^8^^,^^9^ making these molecules promising targets for mechanistic investigation. Within this broad category, several classes of signalling lipids are involved in these processes. Oxylipins, including eicosanoids as a major subclass, play central roles in inflammatory signalling and vascular regulation,^10^^,^^11^ while lysophospholipids are known to modulate endothelial function.^12^

However, critical knowledge gaps remain regarding the precise role of signalling lipids in HFpEF. Most existing studies have focused on conventional blood lipids (e.g., triglycerides and cholesterol) or general metabolic disturbances in HF, without systematically characterising the specific changes in signalling lipid profiles that occur in HFpEF.^13^^,^^14^ Additionally, no studies have investigated the metabolic changes from the pre-clinical to the established phase of HFpEF.

To address these limitations, we aimed to systematically investigate alterations in signalling lipids in incident HFpEF using targeted metabolomics. The lipid perturbations identified through this approach will enhance understanding of metabolic changes during HFpEF progression and provide insights for the early diagnosis of HFpEF and advancements in its therapeutic management.

## Methods

### Study design

This research used data and plasma samples from the Prevention of Renal and Vascular End-stage Disease (PREVEND) study, a community-based cohort in Groningen, the Netherlands. Between 1997 and 1998, all residents aged 28–75 years in the city of Groningen were invited to participate and 8592 participants were enrolled and underwent up to five study visits between 1997 and 2011. These study visits correspond to the PREVEND screening rounds, with the second through fifth rounds conducted between 2001 and 2012. The PREVEND study was designed to investigate associations of micro-albuminuria (urinary albumin excretion ≥10 mg/L) with incident cardio-renal disease.^15^ Participants were followed through linkage and review of hospital records for incident clinical outcomes.

### Study population

For the current lipidomics analysis, we included EDTA plasma samples collected at the second through fifth PREVEND screening rounds (2001–2012). Participants were classified into three groups: (i) control participants without a diagnosis of HF during follow-up (Control; 172 plasma samples from 86 individuals), (ii) participants who later developed HFpEF with plasma collected prior to their HFpEF diagnosis (PreDx-HFpEF; 125 plasma samples from 82 individuals), and (iii) a subset of participants with an additional plasma sample collected after HFpEF diagnosis (PostDx-HFpEF; 30 plasma samples from 30 individuals), paired to their corresponding pre-diagnostic sample from the same individual. After the second PREVEND screening round, 88 participants developed HFpEF during follow-up.^16^ In the present study, 82 cases contributed pre-diagnostic samples due to plasma availability and analytical inclusion criteria (e.g., sufficient sample volume and quality control). Accordingly, for this metabolomics study inclusion was restricted by plasma availability, which may introduce selection related to sample accessibility. No formal sample size calculation was performed, and all eligible participants with available plasma samples and required clinical data in PREVEND were included. Post-diagnostic analyses were considered exploratory due to the limited number of PostDx-HFpEF samples.

### Clinical data collection

During each PREVEND screening round, participants underwent a standardised clinical examination at the outpatient clinic. Data on medical history, medication use, and lifestyle factors were collected using structured questionnaires. Anthropometric measurements, including height and weight, were obtained following standardised procedures. Blood pressure was measured in the supine position using an automated device after a period of rest. Fasting blood samples were obtained and urine was collected for laboratory analyses, including routine clinical chemistry and biomarker measurements. All laboratory analyses were performed in a central laboratory according to standardised protocols.

### Ascertainment of HFpEF events

Incident heart failure events were identified by screening hospital records from University Medical Center Groningen and Martini Hospital. Suspected cases were evaluated by an independent endpoint adjudication committee; each potential event was validated by two experts through review of available clinical information. HFpEF was defined as clinically adjudicated heart failure with a left ventricular ejection fraction (LVEF) ≥ 50% at the time of diagnosis.

### Signalling lipids profiling

A 50 μL aliquot of EDTA plasma was used for metabolomics analysis. Signalling lipids were profiled using a previously established LC-MS/MS method,^17^ which targets a total of 261 analytes, including free fatty acids (omega-3, omega-6, omega-9), free oxylipins (isoprostanes, prostaglandins, and other oxidised lipids), lysophospholipids, sphingolipids, endocannabinoids, and bile acids. Detailed methodological information, including limits of detection and quantification (LOD/LOQ), calibration characteristics, analytical reproducibility, analyte stability, chromatographic separation of lipid species, and mass spectrometric acquisition parameters, as well as validation using NIST reference plasma materials, is described in the same publication. In addition, the steroid cluster was specifically quantified using an in-house developed assay (Protocol S1). In the present study, only the non-esterified fraction of signalling lipids in plasma was quantified using our targeted LC-MS/MS platform, whereas esterified oxylipins incorporated in complex lipid assemblies (e.g., phospholipids or lipoproteins) were not measured.

Following data acquisition from the two analytical methods, raw data were processed using Sciex OS software (AB SCIEX, Version 2·1·6). Relative concentrations were calculated as the ratio of the analyte peak area to that of its assigned internal standard. Data quality control was performed using an in-house developed mzQuality workflow,^18^ resulting in 182 reliably detected metabolites (Table S1). Furthermore, sums of metabolites within each lipid subclass and ratios of specific targets to their precursor molecules were computed. This yielded a total of 247 variables for subsequent statistical analyses, comprising 182 detectable signalling lipids, two lipid subclass sums, and 63 lipid ratios (Table S2).

### Statistical analyses

Baseline clinical characteristics were compared among the Control, PreDx-HFpEF, and PostDx-HFpEF groups. Pairwise group differences were evaluated using Fisher's exact tests for categorical variables and Student's t-tests for continuous variables after confirming the homogeneity of variance. Missingness rates for each clinical variable are shown in Table S3.

Prior to analyses of lipid targets, the relative concentrations of all signalling lipids were log_2_-transformed and centre-scaled to achieve a normal distribution and ensure comparability across targets. Metabolites with a missing rate exceeding 20% within each group were excluded. After exclusion, the distribution of metabolites across different missing rate ranges was as follows: 212 metabolites with <5% missing values, 18 with a missing rate of 6%–10%, and seven with a missing rate of 11%–20%. Missing values were attributed to low metabolite concentrations in samples. Therefore, missing values were imputed as one-tenth of the minimum observed value for the respective metabolite. Spearman correlation analysis was applied to identify potential clinical covariates associated with signalling lipids, as several clinical variables were categorical or ordinal.

The analytical strategy was designed to characterise signalling lipid alterations across different stages of HFpEF and to distinguish global lipid pattern changes from stage-specific associations. First, unsupervised multivariate analyses were applied to assess overall differences in signalling lipid profiles between groups, providing a global view of lipidomic variation without prespecified hypotheses. Second, Cox proportional-hazards models were used to identify signalling lipids associated with incident HFpEF in the pre-diagnostic phase, explicitly incorporating time-to-event information to capture early predictive associations. Third, logistic regression models were employed to evaluate cross-sectional differences after HFpEF diagnosis and to assess changes between pre- and post-diagnostic states, thereby focussing on disease-state–related lipid alterations. Finally, complementary visualisation and correlation analyses were used to integrate findings across methods and to support biological interpretation.

Principal component analysis (PCA) was conducted to characterise signalling lipid profiles across different groups. In addition, permutational multivariate analysis of variance (PERMANOVA) was used to evaluate group dissimilarities. The analysis was performed on Euclidean distance matrices derived from metabolite intensities, with significance determined by permutation testing. Pairwise group comparisons were conducted, and homogeneity of within-group dispersions was assessed prior to interpretation. A heatmap was generated to visualise the top 50 metabolites contributing to the PCA results.

Cox proportional-hazards models were used to compare the Control and PreDx-HFpEF groups during the pre-diagnostic phase. For the post-diagnostic phase, logistic regression models were applied to compare the Control, PreDx-HFpEF, and PostDx-HFpEF groups. In both Cox and logistic regression analyses, models were adjusted for age, sex, diabetes status, and the use of lipid-lowering drugs, non-steroidal anti-inflammatory drugs (NSAIDs), and antidiabetic drugs. Participant IDs were incorporated as random effects to account for multiple samples contributed by individual participants for both Cox and logistic regression models.

For Cox regression analyses, the outcome was time to incident HFpEF, defined as the interval from baseline sample collection to the date of HFpEF diagnosis. Participants without HFpEF were censored at the date of last follow-up. Each lipid metabolite or lipid ratio was evaluated as the predictor in a separate Cox model comparing Control and PreDx-HFpEF groups. The Cox model was specified as: $h_{i}\left(t\right){=h}_{0}\left(t\right)\;\exp\left(\beta_{1}\times {lipid}_{i}+\beta_{2}\times {covariates}_{i}+b_{i}\right)$ where $h_{i}\left(t\right)$ is the hazard of HFpEF for participant i at time t, and $h_{0}\left(t\right)$ is the baseline hazard function capturing the time dependence. The term $b_{i}$ is a participant-specific random intercept. The proportional hazards assumption was assessed, and no substantial violations were observed for the lipid predictors. In addition, time-dependent receiver operating characteristic (ROC) analyses based on Cox regression models were conducted to evaluate the predictive value of lipid targets within specific pre-diagnostic time windows, with time intervals calculated using sample collection and diagnosis dates, and discrimination performance was quantified using the area under the curve (AUC).

Logistic regression models were applied for cross-sectional comparisons between Control and PostDx-HFpEF groups and between PreDx-HFpEF and PostDx-HFpEF groups, with HFpEF status as the binary outcome. All PostDx-HFpEF samples had corresponding PreDx-HFpEF samples from the same individuals, whereas the PreDx-HFpEF group additionally included samples from individuals without post-diagnostic samples. Therefore, all available samples were included in the analysis to maximise statistical power. Each lipid metabolite or lipid ratio was evaluated as the predictor in a separate model. The logistic mixed-effects model was specified as: $logit\left\{P{\left(HFpEF\right)}_{i}=1\right\}=\alpha +\beta_{1}\times {lipid}_{i}+\beta_{2}\times {covariates}_{i}+b_{i}$ where $P{\left(HFpEF\right)}_{i}=1$ is the probability of HFpEF and $b_{i}$ is a subject-specific random intercept. Furthermore, logistic regression model-based ROC analyses were performed to assess the discriminative performance of significant lipid targets identified from logistic regression models. In addition, individual clinical variables were also evaluated using separate logistic regression models to assess their associations with HFpEF status. These analyses were performed without adjustment for additional covariates.

Significant metabolites that appeared in at least two of the comparisons were further inspected. To account for potential confounders, metabolite concentrations were adjusted using linear mixed-effects models. The models included age, sex, diabetes status, lipid-lowering medication, NSAID use, and antidiabetic medication use as fixed effects, with participant ID included as a random effect. Adjusted metabolite levels were derived by adding the residuals from these models to the overall mean concentration of each metabolite. Group differences were assessed using Tukey's HSD tests.

Pearson partial correlation analyses were performed to examine relationships between HDL and significant signalling lipids identified by logistic regression models (PreDx-HFpEF vs PostDx-HFpEF), as both HDL and signalling lipids are continuous. Mediation analyses were conducted to evaluate whether HDL mediates the association between HFpEF phase (PreDx-HFpEF vs PostDx-HFpEF) and signalling lipid targets, with HFpEF phase as the exposure, HDL as the mediator, and each signalling lipid target as the outcome.

All the p-values were adjusted using the Benjamini–Hochberg false discovery rate (FDR) method, resulting in adjusted p-values (p.adj). A p.adj ≤ 0.05 was considered statistically significant. All the statistical analyses in this study were conducted using R (version 4·5·0)

### Ethics

The PREVEND study was approved by the Medical Ethics Committee of the University Medical Center Groningen and conducted in compliance with the Declaration of Helsinki (approval number: MEC96/01/022). Written informed consent was obtained from all participants.

### Role of the funding source

Funders did not participate in the study design, data collection, data analyses, interpretation, or writing of the manuscript.

## Results

### Baseline characteristics

Table 1 summarises the baseline clinical characteristics of the Control, PreDx-HFpEF, and PostDx-HFpEF groups. Overall, the PostDx-HFpEF group exhibited a higher burden of cardiometabolic comorbidities, greater use of cardiovascular medications, and more advanced renal impairment compared with the other two groups (Table 1; Figure S1).

**Table 1:** Participant characteristics.

Characteristic	Control (n = 172)	PreDx-HFpEF (n = 125)	PostDx-HFpEF (n = 30)
Female sex (n)	88 (51%)	69 (55%)	13 (43%)
Age (years)	69.1 ± 7.6	68.8 ± 7.5	72.8 ± 6.4^b^^,^^c^
BMI (kg/m^2^)	28.9 ± 4.4	29.8 ± 5.9	29.3 ± 4.5
Systolic blood pressure (mmHg)	137.3 ± 19.3	142.5 ± 25.6^a^	139.7 ± 24.3
Diastolic blood pressure (mmHg)	74.7 ± 8.8	74.5 ± 10.1	72.8 ± 10.3^b^
Pulse (beats per minute)	68.6 ± 9.6	66 ± 12.7^a^	63.8 ± 10.8^b^
HDL (mmol/L)	1.3 ± 0.4	1.3 ± 0.3	1.5 ± 0.3^c^
Cholesterol (mmol/L)	5.3 ± 1.2	5.2 ± 1.0	4.6 ± 1.0^b^^,^^c^
Glucose (mmol/L)	5.5 ± 1.3	5.8 ± 1.8	6.1 ± 1.4^b^
eGFR (Creatinine-Cystatin C)	76.6 ± 17.2	71.5 ± 16.3^a^	67.3 ± 21.5^b^
Urinary Albumin-Creatinine Ratio (mg/mmol)	4.7 ± 9.0	11.6 ± 39.6^a^	22.3 ± 80.7^b^
CKD stage (n)			
a. Stage 1	39 (23%)	19 (15%)	3 (10%)
b. Stage 2	98 (57%)	76 (61%)	15 (50%)
c. Stage 3	24 (14%)	25 (20%)^a^	5 (17%)
d. Stage 4	2 (1%)	0 (0%)	1 (3%)
e. Stage 5	0 (0%)	0 (0%)	1 (3%)
Cerebrovascular event in the past (n)	8 (5%)	0 (0%)^b^	0 (0%)
Peripheral event in the past (n)	3 (2%)	0 (0%)	0 (0%)
Cardiovascular event in the past (n)	35 (20%)	49 (39%)	9 (30%)
Venous thromboembolism in the past (n)	3 (2%)	5 (4%)	1 (3%)
Hypertension (n)	67 (39%)	71 (57%)^a^	27 (90%)^b^^,^^c^
Hyperlipidaemia (n)	50 (29%)	40 (32%)	19 (63%)^b^^,^^c^
Diabetes (n)	15 (9%)	16 (13%)	8 (27%)
ACEi/ARB use (n)	32 (19%)	41 (33%)	22 (73%)
Lipid-lowering drug use (n)	42 (24%)	34 (27%)	13 (43%)^b^^,^^c^
Oral antidiabetic drug use (n)	12 (7%)	16 (13%)	6 (20%)
Beta blocker use (n)	21 (12%)	46 (37%)^a^	15 (50%)^b^
Insulin use (n)	1 (1%)	4 (3%)	2 (7%)
NSAID use (n)	23 (13%)	19 (15%)	3 (10%)
Renin inhibitors use (n)	0 (0%)	0 (0%)	0 (0%)

Data are presented as mean ± SD or n (%).

BMI, body mass index; HDL, high density lipoprotein; CKD, chronic kidney disease; ACEi, angiotensin-converting enzyme inhibitors; ARB, angiotensin receptor blockers; NSAID, non-steroidal anti-inflammatory drugs.

Data in Table 1 are presented at the plasma sample level and reflect clinical characteristics recorded at the time of each sample collection, including repeated samples from the same individuals across different PREVEND screening visits. Significance is indicated as follows:

a p.adj < 0.05 for Control vs. PreDx-HFpEF.

b p.adj < 0.05 for PostDx-HFpEF vs. Control.

c p.adj < 0.05 for PostDx-HFpEF vs. PreDx-HFpEF.

Notably, a range of clinical variables were identified to be associated with signalling lipids (Figure S2). Among these, HDL and total cholesterol are indicators of lipid metabolism. Several kidney function parameters exhibited associations with lipid targets; however, such relationships lack clear mechanistic links. Consequently, these kidney function indicators were not included as adjustment factors for the lipid targets. Similarly, commonly prescribed antihypertensive medications, including renin inhibitors, RAAS inhibitors, and beta-blockers, were associated with only a small proportion of detected signalling lipids and are not known to directly regulate lipid metabolism. Consequently, these medications were not considered as covariates. Instead, variables with evident causal relationships to the lipid targets were selected as covariates, including age, sex, diabetes status, and the use of lipid-lowering agents, non-steroidal anti-inflammatory drugs (NSAIDs), and antidiabetic medications.

### Multivariate analyses showed differences in signalling lipid profiles

**Fig. 1:**
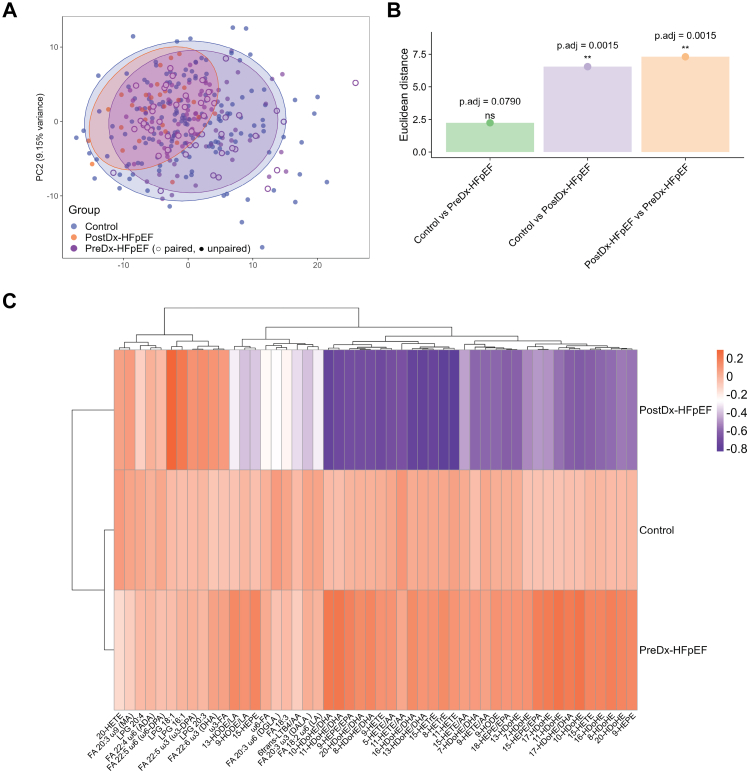
**Multivariate analysis of signalling lipids across Control, PreDx-HFpEF, and PostDx-HFpEF groups.** (A) Principal component analysis (PCA) of metabolomics data. (B) Intergroup metabolic dissimilarity assessed using Euclidean distance. (C) Heatmap displaying the top 50 contributing metabolites, plotted by group mean. ∗∗p.adj < 0.01. The p.adj values were derived from pairwise PERMANOVA with FDR correction. Data were obtained from 172 control, 125 PreDx-HFpEF, and 30 PostDx-HFpEF samples.

Multivariate analyses showed differences in signalling lipid profiles among the Control, PreDx-HFpEF, and PostDx-HFpEF groups. PCA demonstrated partial separation of PostDx-HFpEF samples from the other two groups, while PreDx-HFpEF samples overlapped substantially with the Control group (Fig. 1A). To avoid potential sample bias, given that PostDx-HFpEF samples were collected from the same participants who also contributed PreDx-HFpEF samples, these paired samples were specifically labelled. No sample separation was observed between paired and unpaired samples. Furthermore, PERMANOVA confirmed significant differences in PostDx-HFpEF compared to the other groups (Fig. 1B). Heatmap analysis (Fig. 1C) highlighted most non-esterified oxylipins as slightly elevated in PreDx-HFpEF but markedly reduced in PostDx-HFpEF, with only a few lysophospholipids and fatty acids increased.

### Elevated non-esterified oxylipins and lysophospholipids in PreDx-HFpEF predict HFpEF incidence

**Fig. 2:**
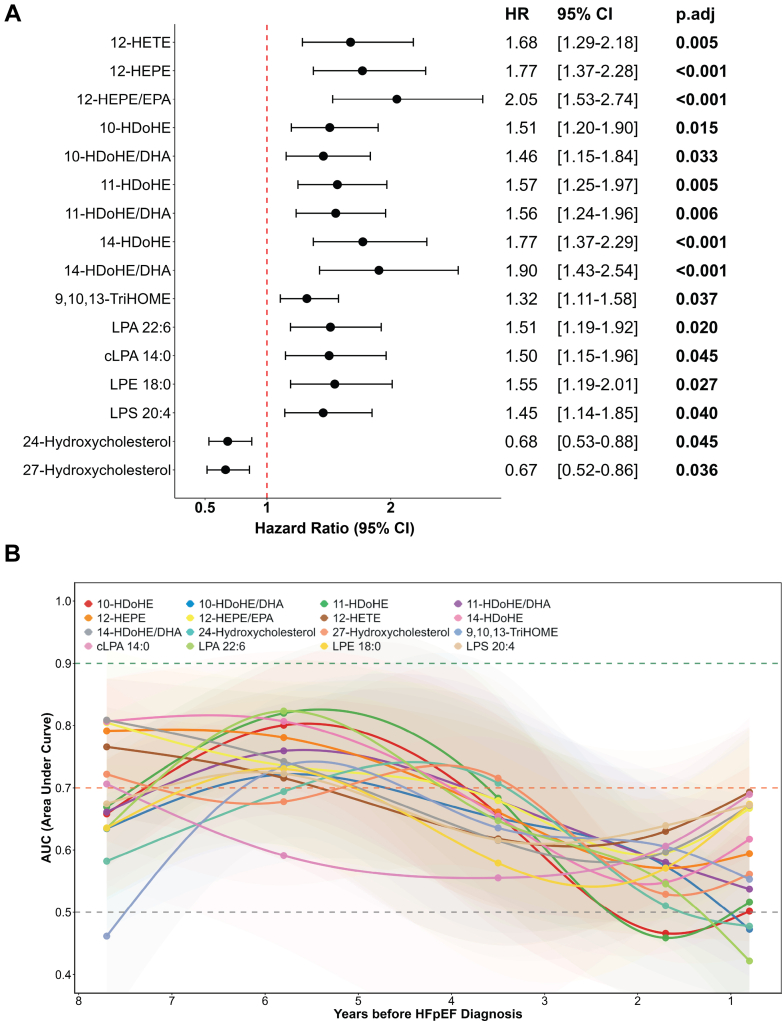
**Significant metabolites distinguishing the Control and PreDx-HFpEF groups.** (A) Forest plot illustrates Cox proportional hazards model results comparing the Control and PreDx-HFpEF groups. (B) Time-dependent ROC analysis of significant metabolites. Hazard ratios represent hazards of developing HFpEF per 1-SD increase in relative abundance of lipid targets. The p.adj values were derived from Cox proportional-hazards models with FDR correction. Data were obtained from 172 control and 125 PreDx-HFpEF samples.

The potential predictors among signalling lipids for HFpEF were identified by comparing control to PreDx-HFpEF samples using Cox proportional hazards models (Fig. 2A). Specifically, ten non-esterified oxylipins (including ratios of oxylipins to their precursors) and four lysophospholipids were found to be positive predictors of HFpEF incidence, while two hydroxycholesterols acted as negative predictors. Among these non-esterified oxylipins, 12-HETE is derived from arachidonic acid (AA); 12-HEPE is derived from eicosapentaenoic acid (EPA); 10-HDoHE, 11-HDoHE, and 14-HDoHE are derived from docosahexaenoic acid (DHA); and 9,10,13-TriHOME is derived from linoleic acid (LA). For relevant clinical variables, none achieved statistical significance following FDR correction. Only age and beta blocker use yielded p-values <0.05 (Figure S3). For the other two lipid indicators, cholesterol and HDL, their hazard ratios were 1.18 and 0.64, respectively.

Furthermore, the predictive potential of these signalling lipids varied over time (Fig. 2B). Overall, the AUC values exhibited a decreasing trend over time. Specifically, 14-HDoHE, 12-HEPE, and their ratios to their respective precursors showed strong discriminative ability approximately 8 years prior to diagnosis, with AUCs around 0.8. For most other significant metabolites, the highest time-dependent AUCs were observed approximately 6 years before diagnosis. Within this time window, 10-HDoHE, 11-HDoHE, 14-HDoHE, and lysophosphatidic acid (LPA) 22:6 also exhibited AUCs of approximately 0.8. Between 6 and 2 years prior to diagnosis, the time-dependent AUCs of these metabolites generally decreased, except for 24-hydroxycholesterol and 27-hydroxycholesterol. One year before diagnosis, several metabolites showed a modest increase in AUC values.

### Signalling lipid profiles in PostDx-HFpEF differed from Control and PreDx-HFpEF

**Fig. 3:**
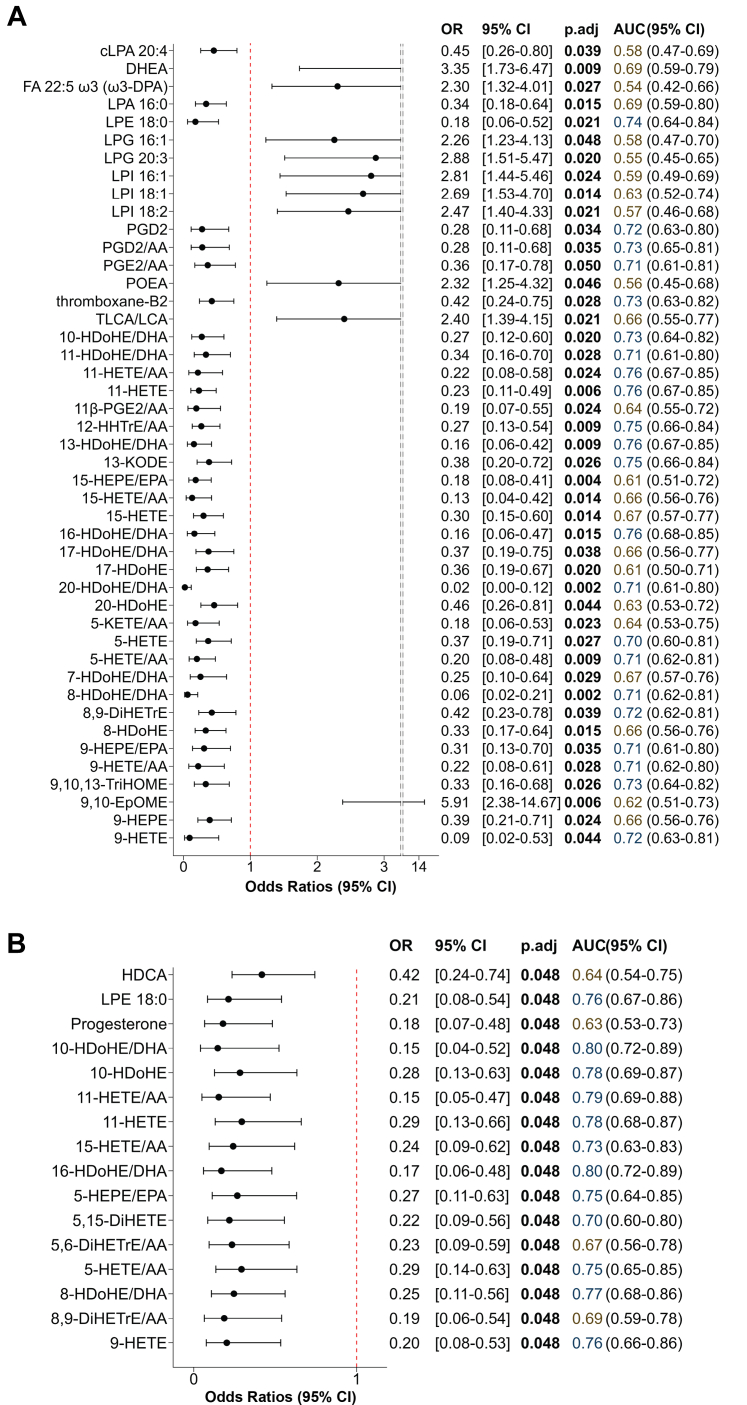
**Significant metabolites distinguishing Control and PreDx-HFpEF groups from the PostDx-HFpEF group.** Forest plots display logistic regression results for two comparisons: (A) Control vs PostDx-HFpEF and (B) PreDx-HFpEF vs PostDx-HFpEF. Odds ratios represent the change in odds of having HFpEF per 1-standard deviation increase in the relative abundance of lipid targets. For AUC values, a brown colour was assigned to those ranging from 0.5 to 0.7, whereas a blue colour was designated for values greater than 0.7. The p.adj values were derived from logistic regression models with FDR correction. Data were obtained from 172 control, 125 PreDx-HFpEF, and 30 PostDx-HFpEF samples.

Logistic regression models revealed significant alterations in signalling lipid profiles in PostDx-HFpEF. As presented in Fig. 3A, non-esterified oxylipin concentrations after adjusting for age, sex, diabetes status, and the use of lipid-lowering drugs, non-steroidal anti-inflammatory drugs (NSAIDs), and antidiabetic drugs were generally associated with reduced odds (odds ratio <1) of PostDx-HFpEF, a trend opposite to that observed in comparisons between Control and PreDx-HFpEF groups. Specifically, 33 non-esterified oxylipins and the ratios of oxylipins to their precursors exhibited significant differences between the Control and PostDx-HFpEF groups. Among these 33 targets, 16 were derived from AA, including HETEs, KETEs, DiHETrEs, PGX2, and thromboxane-2; 11 originated from DHA (HDoHEs); three were derived from EPA (HEPEs); and three came from LA (KODE, EpOME, and TriHOME). Consistent findings were also observed when comparing the PreDx-HFpEF and PostDx-HFpEF groups. Specifically, eight of the aforementioned non-esterified oxylipins exhibited a consistent trend of lower odds of progressing to PostDx-HFpEF (Fig. 3B). These include the AA-derived 5-HETE, 9-HETE, 11-HETE, 15-HETE and 8,9-DiHETrE, DHA-derived 8- HDoHE, 10- HDoHE and 16-HDoHE. Notably, 9,10-EpOME was the only non-esterified oxylipin that showed higher odds ratio (>1) in PostDx-HFpEF when compared to the Control group (Fig. 3A). Regarding lysophospholipids, specific species exhibited differential odds ratios when comparing PostDx-HFpEF group to Control group. Lysophosphatidylethanolamine (LPE) 18:0, lysophosphatidic acid (LPA) 16:0, and cyclic LPA (cLPA) 20:4 all exhibited odds ratios of less than 1, whereas two lysophosphatidylglycerol (LPG) species (16:1, 20:3) and three lysophosphatidylinositol (LPI) species (16:1, 18:1, 18:2) showed odds ratios greater than 1.

For clinical variables, the presence of hypertension, hyperlipidaemia, and related drug use were strong indicators of HFpEF (Table 2). While no significant differences were observed in blood pressure, its odds ratio was close to 1. Furthermore, higher cholesterol levels were associated with reduced likelihood of being classified into the PostDx-HFpEF group; conversely, higher HDL levels were positively associated with HFpEF (Table 2). Notably, the associations of cholesterol and HDL with PostDx-HFpEF were also opposite to those with PreDx-HFpEF.

**Table 2:** Odds ratios of clinical variables from logistic regression models.

Indicators	Control vs PostDx-HFpEF				PreDx-HFpEF vs PostDx-HFpEF			
	Odds ratio	95% CI	p	p.adj	Odds ratio	95% CI	p	p.adj
Sex	0.66	[0.29–1.54]	0.339	0.448	0.60	[0.26–1.39]	0.233	0.480
Age	1.08	[1.01–1.15]	**0.024**	0.088	1.09	[1.02–1.16]	**0.012**	0.100
BMI	1.02	[0.93–1.12]	0.620	0.731	0.99	[0.91–1.07]	0.723	0.804
Systolic blood pressure	1.00	[0.99–1.02]	0.659	0.737	0.99	[0.98–1.01]	0.520	0.650
Diastolic blood pressure	0.98	[0.94–1.03]	0.382	0.485	0.98	[0.94–1.03]	0.457	0.603
Pulse	0.95	[0.91–0.99]	**0.029**	0.092	0.98	[0.95–1.02]	0.364	0.585
Cerebrovascular event	3.15	[0.74–13.51]	0.122	0.232	1.25	[0.35–4.4]	0.731	0.804
Cardiovascular event	1.62	[0.63–4.14]	0.314	0.448	0.71	[0.29–1.73]	0.446	0.603
Cigarettes in the present	0.22	[0.03–1.77]	0.155	0.255	0.11	[0.01–0.88]	**0.038**	0.138
Numbers of cigarettes per day	3.56	[0.62–20.43]	0.155	0.255	1.76	[0.3–10.47]	0.532	0.650
Cigarettes in the past	1.15	[0.46–2.89]	0.767	0.791	1.48	[0.58–3.76]	0.408	0.585
Years of smoking in the past	1.08	[0.05–21.29]	0.959	0.959	0.80	[0.04–17.2]	0.887	0.898
Alcoholic beverages	1.30	[0.37–4.56]	0.684	0.737	1.17	[0.33–4.15]	0.807	0.859
Frequency of exercise	1.48	[0.38–5.76]	0.568	0.694	2.07	[0.53–7.99]	0.293	0.536
Frequency of sports	0.52	[0.14–1.97]	0.336	0.448	0.56	[0.14–2.15]	0.394	0.585
HDL (mmol/L)	2.70	[0.81–9.05]	0.106	0.220	4.12	[1.09–15.61]	**0.038**	0.138
**Cholesterol (mmol/L)**	0.55	[0.36–0.82]	**0.004**	**0.026**	0.57	[0.37–0.9]	**0.016**	0.104
Glucose (mmol/L)	1.36	[0.99–1.86]	0.055	0.138	1.11	[0.88–1.41]	0.377	0.585
Serum Cystatin C (mg/L)	4.24	[1.12–15.97]	**0.033**	0.092	3.03	[0.86–10.73]	0.086	0.236
Serum creatinine (mg/dL)	2.93	[0.82–10.45]	0.098	0.216	2.20	[0.76–6.39]	0.148	0.377
Urine creatinine (mmol/L)	0.87	[0.73–1.04]	0.127	0.232	0.89	[0.74–1.07]	0.208	0.458
Urine albumin (mg/L)	1.00	[1.00–1.01]	0.171	0.269	1.00	[1–1]	0.388	0.585
eGFR (Creatinine-Cystatin C)	0.97	[0.95–1.00]	**0.033**	0.092	0.99	[0.96–1.01]	0.267	0.518
CKD stage	2.00	[0.52–7.68]	0.313	0.448	1.10	[0.27–4.42]	0.898	0.898
**Hypertension**	15.19	[4.26–54.10]	**2.70E-05**	**2.97E-04**	6.70	[1.88–23.88]	**0.003**	**0.037**
**Hyperlipidaemia**	4.73	[1.96–11.44]	**5.60E-04**	**4.62E-03**	3.72	[1.55–8.94]	**0.003**	**0.037**
Diabetes	3.63	[1.22–10.82]	**0.021**	0.086	2.78	[0.97–7.97]	0.058	0.191
**ACEi/ARB use**	44.38	[9.35–210.68]	**1.82E-06**	**6.00E-05**	19.96	[4.36–91.45]	**1.15E-04**	**3.81E-03**
Lipid-lowering drug use	3.28	[1.26–8.54]	**0.015**	0.070	2.90	[1.13–7.45]	**0.027**	0.127
Oral antidiabetic drug use	3.23	[0.96–10.87]	0.059	0.138	2.08	[0.67–6.44]	0.204	0.458
**Beta blocker use**	10.86	[3.81–30.97]	**8.17E-06**	**1.35E-04**	2.38	[0.93–6.11]	0.070	0.210
**Statins use**	3.72	[1.44–9.64]	**0.007**	**0.038**	3.01	[1.17–7.72]	**0.022**	0.121
NSAID use	0.76	[0.20–2.94]	0.692	0.737	0.71	[0.19–2.75]	0.625	0.736

Values of p < 0.05 or p.adj < 0.05 were highlighted. The p and p.adj values were derived from logistic regression models without or with FDR correction, respectively. The study involved 172 control, 125 PreDx-HFpEF, and 30 PostDx-HFpEF samples.

### Signalling lipids variation is independent of HDL

**Fig. 4:**
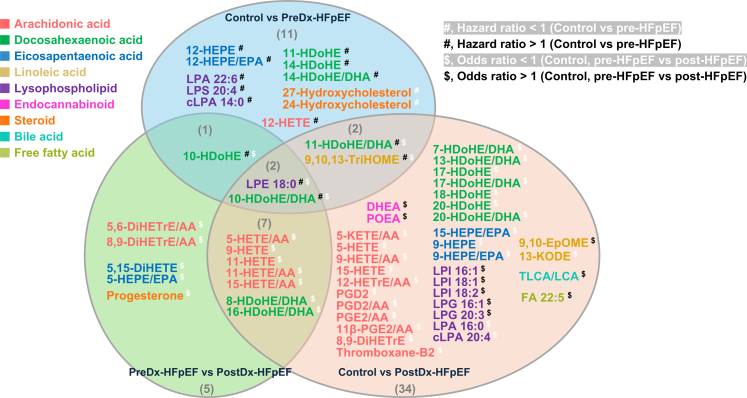
**Significant metabolites by Cox models and logistic regression models.** In Cox models, metabolites with a HR < 1 were marked with a white hashtag, while those with a HR > 1 were marked with a black hashtag. In logistic regression models, metabolites with an OR < 1 were marked with a white dollar sign, and those with an OR > 1 were marked with a black dollar sign.

**Fig. 5:**
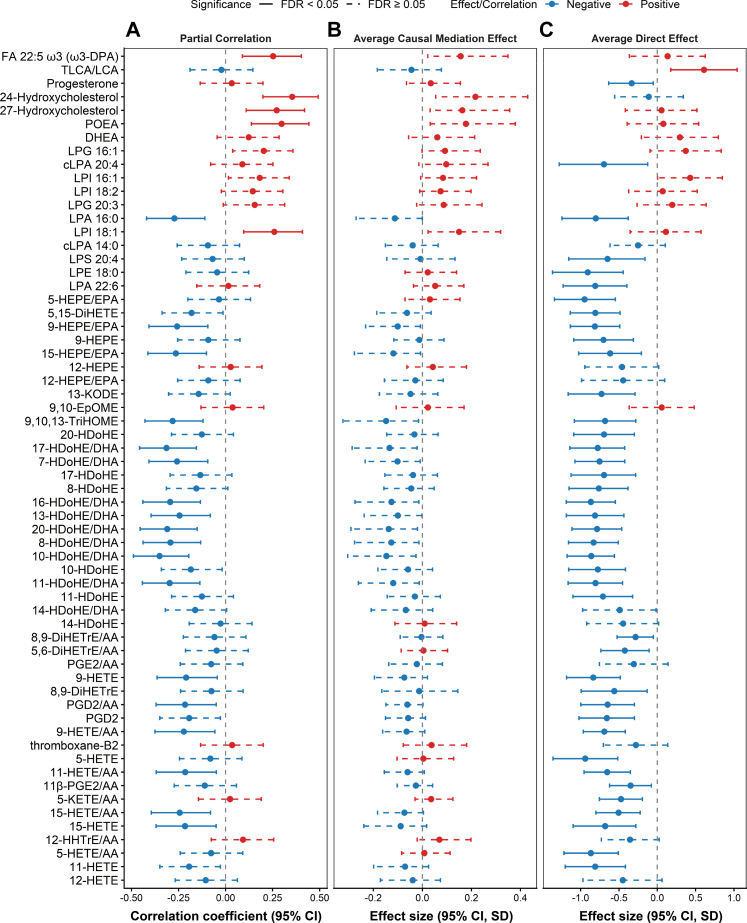
**Associations between significant lipids and HDL.** (A) Pearson partial correlation between significant lipids and HDL. (B) Average causal mediation effects and (C) average direct effects between significant lipids and HDL, with HDL serving as the mediator and group as the causal factor. Solid lines indicate statistical significance, and dashed lines indicate non-significance. Red denotes positive effects or correlations, and blue denotes negative effects or correlations. The p.adj values were derived from Pearson partial correlation analyses and mediation analyses with FDR correction. Data were obtained from 125 PreDx-HFpEF and 30 PostDx-HFpEF samples.

Non-esterified oxylipins and lysophospholipids emerged as the primary lipid classes exhibiting significant variations across different comparative analyses after adjustment of age, sex, diabetes status, and the use of lipid-lowering drugs, NSAIDs, and antidiabetic drugs (Fig. 4). From the PreDx to PostDx stage, both HDL and several signalling lipids exhibited changes in the opposite direction. Therefore, the associations between HDL and signalling lipids were inspected. Pearson partial correlation analysis showed that 23 of 62 significant signalling lipids correlated with HDL (Fig. 5A). Among these, only FA 22:5, LPI 18:1, and 24,27-hydroxycholesterol were positively correlated, while the remaining 19 non-esterified oxylipins displayed negative correlations. Further mediation analysis confirmed that variations of all 62 signalling lipids were not mediated by HDL, as no significant average causal mediation effects were observed (Fig. 5B). Instead, as HFpEF progressed, variations in 42 (68%) of the significant signalling lipids were independent of HDL (Fig. 5C). Specifically, DHA-derived HDoHEs, EPA-derived HEPEs, and AA-derived HETEs accounted for the negative associations with HFpEF progression, whereas only TLCA/LCA showed a positive association.

### Consistently significant signalling lipids across all comparisons: 10-HDoHE/DHA and LPE 18:0

**Fig. 6:**
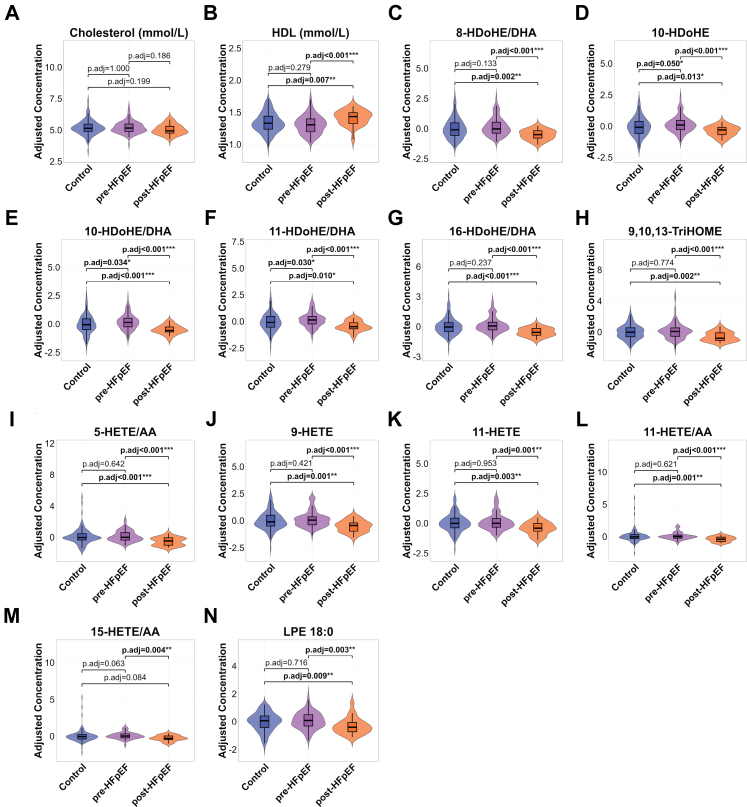
**Violin plots of cholesterol, HDL and significant metabolites.** Cholesterol (A) and HDL (B) were adjusted for lipid-lowering drugs. Plotted metabolites are those identified as significant targets in at least two comparisons, including: (C) 8-HDoHE/DHA, (D) 10-HDoHE, (E) 10-HDoHE/DHA, (F) 11-HDoHE/DHA, (G) 16-HDoHE/DHA, (H) 9,10,13-TriHOME, (I) 5-HETE/AA, (J) 9-HETE, (K) 11-HETE, (L) 11-HETE/AA, (M) 15-HETE/AA and (N) LPE 18:0. ∗p.adj < 0.05; ∗∗p.adj < 0.01; ∗∗∗p.adj < 0.001. The p.adj values were derived from Dunn's tests with FDR correction. Data were obtained from 172 control, 125 PreDx-HFpEF, and 30 PostDx-HFpEF samples.

Significant signalling lipids overlapping across all comparisons were specifically plotted (Fig. 6). Notably, metabolites derived from DHA and AA, specifically HDoHEs and HETEs, along with LA-derived 9,10,13-TriHOME and LPE 18:0, consistently displayed similar trends. All these signalling lipid species were significantly lower in the PostDx-HFpEF group. In contrast, the PreDx-HFpEF group exhibited a slight upward trend, with only 10-HDoHE and 11-HDoHE reaching statistical significance. This observation aligns with previous findings from Cox proportional hazards models (Fig. 2).

Cholesterol and HDL, key indicators of lipid metabolism, also distinguished the study groups. While cholesterol did not retain significance after adjustment for lipid-lowering drug use, it was significantly lower in the PostDx-HFpEF group prior to the adjustment (Figure S4). In contrast, HDL levels were significantly elevated in the PostDx-HFpEF group both before and after adjustment.

Furthermore, paired t-test analyses confirmed consistent changes in the majority of lipid targets, except for 5-HETE/AA and 9-HETE, as well as HDL, within individuals not receiving lipid-lowering therapy (Figure S5), suggesting that these patterns were unlikely to be driven by lipid-lowering medication use. In addition, given that many of the identified oxylipins are derived from polyunsaturated fatty acids (PUFAs), we examined the circulating levels of their precursor fatty acids, including DHA, AA and EPA. No significant differences in the levels of these precursor fatty acids were observed between groups (Figure S6).

## Discussion

This study identifies stage-specific alterations in signalling lipids across HFpEF development. First, although overall signalling lipid profiles were largely similar between PreDx-HFpEF individuals and controls, a few signalling lipids and lipid ratios showed significant associations with future HFpEF onset when time-to-event information was considered, suggesting subtle metabolic alterations preceding disease diagnosis. Second, after HFpEF diagnosis, these lipid signatures were attenuated, indicating a shift in disease-associated metabolic patterns. Third, oxylipins, particularly those derived from DHA and AA, emerged as a key cluster of signalling lipids involved in HFpEF progression.

Of note, 43% (44/103) of detected non-esterified oxylipins exhibited statistical significance across different comparisons, and their variations are independent of HDL. These oxylipins play a crucial role in the progression of HFpEF, and the metabolic pathways they involve as well as their functions in the cardiovascular system are detailed in Figure S7.

Furthermore, the time-dependent predictive performance observed in the present study warrants further clarification. The variation in AUC values over time reflects changes in the discriminative ability of specific signalling lipids to distinguish future HFpEF cases from controls within defined pre-diagnostic windows. HFpEF is increasingly recognised as a heterogeneous syndrome characterised by early low-grade inflammation and endothelial dysfunction that may precede clinical diagnosis.^19^^,^^20^ During this early pre-clinical phase, PUFA-derived oxylipins and related signalling lipids, which are closely linked to inflammatory and vascular pathways, may more sensitively capture these initial pathophysiological disturbances, resulting in higher predictive performance.^21^^,^^22^ As the disease progresses towards clinical diagnosis, we hypothesise that the underlying pathophysiology evolves from systemic inflammatory processes into more complex mechanisms involving multiple systems and regulatory pathways, with increasing impact on cardiac and extracardiac function. This growing biological complexity may dilute the contribution of individual lipid mediators, thereby reducing the specificity and time-dependent predictive performance of single signalling lipid markers.

HDL and cholesterol, two pivotal markers of lipid metabolism, have been identified as key risk factors in HF. In the Framingham Heart Study, participants with elevated baseline cholesterol levels and those with low HDL concentrations exhibited an increased risk of HF,^23^ which aligns with the findings from our Cox model comparing PreDx-HFpEF and Control groups. However, HDL levels differed in the PostDx-HFpEF subgroup compared with earlier disease stages, a finding that has been infrequently reported. These differences may relate to impaired cholesterol efflux capacity and reduced anti-inflammatory properties of HDL observed in patients with HFpEF,^24^ which may trigger compensatory mechanisms.

Fatty acids and their derived oxylipins are widely recognised as critical lipid groups in cardiovascular diseases. In this study, we focused on the circulating non-esterified fraction of oxylipins, which represents a measurable pool of lipid mediators in plasma and has been linked to inflammatory and vascular signalling pathways.^25^ While oxylipins can also be incorporated into complex lipids as esterified forms and influence signalling pathways,^26^ these species were not quantified in the present analysis. Specifically, eicosanoids, oxidised metabolites of AA, DHA, EPA, and LA, are generated through enzymatic pathways involving lipoxygenases (LOX), cyclooxygenases (COX), cytochrome P450 (CYP) enzymes, or non-enzymatic processes. Biosynthetically, 5-LOX, 12-LOX, and 15-LOX catalyse the production of HETEs, HDoHEs, HEPEs, and TriHOMEs from AA, DHA, EPA, and LA, respectively. Prostaglandin synthesis is regulated by COX-1, COX-2, and prostaglandin E synthase (PGES). In addition, CYP enzyme families participate in generating DiHETrEs, and EpOMEs from AA and LA.^8^

These eicosanoids also exhibit diverse cardiovascular bioactivities. Oxylipins derived from AA, a representative omega-6 fatty acid, have frequently been associated with pro-inflammatory and adverse cardiovascular effects, although their biological actions are heterogeneous and context-dependent. 5-, 9-, 11- and 12-HETE can promote leucocyte migration and chemokinesis.^11^ Additionally, 5-, 12- and 15-HETEs induce ventricular cardiomyocyte hypertrophy.^27^ 9-HETE and PGD2 were reported to be predictors of 1-year death in acute decompensated HF.^28^ On the contrary, the omega-3 fatty acids DHA and EPA and their oxylipins were reported to have beneficial cardiovascular effects. Omega-3 fatty acids supplementation could elevate 11- and 20-HDoHE, and improve endothelial function after acute myocardial infarction.^29^ In other myocardial infarction research, the total of DHA-derived oxylipins including 8-, 10-, 11-, 13-, 16-, 17- and 20-HDoHE were significantly correlated with markers of cardiac injury during ischaemia.^30^ However, the biosynthetic pathway of certain HDoHE species, particularly 10-HDoHE, remains debated, with both enzymatic (e.g., LOX-mediated) and non-enzymatic oxidative pathways proposed.^31^^,^^32^ EPA derived 9,10,13-TriHOME could serve as an oxidative stress marker as it is one of the most significant oxylipins in oxidised LDL.^33^ In the present study, all the non-esterified oxylipins except 9,10-EpOME, exhibited the same trends despite their diverse roles in cardiovascular system, which reflects overall pattern of non-esterified oxylipin variations in HFpEF progression. These eicosanoids identified as positive predictors for HFpEF incidence (Control vs PreDx-HFpEF), later showed a negative association with diagnosed HFpEF (PreDx-HFpEF, Control vs PostDx-HFpEF). This metabolic change was independent of HDL variation, representing manifestations of lipid dysregulation in HFpEF.

9,10-EpOME, one of the leukotoxins, has been reported to induce oxidative stress in vascular endothelial cells. Intravenous administration of 9,10-EpOME has been shown to exert cardiodepressive effects in dogs.^10^ A recent study revealed divergent associations of 9,10-EpOME with HFpEF across two cohorts. In one cohort, it was positively associated with incident HFpEF when comparing the Control group with PreDx-HFpEF group; in contrast, in the other cohort, it was associated with reduced odds of HFpEF when comparing the Control group with the HFpEF group.^34^ The observed contradictory alterations in eicosanoids across different stages of HFpEF progression align with the shifts we identified.

Beyond fatty acids and oxylipins, lysophospholipids also participate in HFpEF progression. The variety of head groups contribute to diverse subclasses of lysophospholipids, such as LPE, LPG, LPA and LPI.^12^ Notably, LPE 18:0 has been linked to chronic stress and an elevated risk of cardiovascular diseases.^35^ LPAs were proven to promote atherosclerosis and increase vessel permeability.^36^ The present study also identified a positive association between LPE 18:0, LPA 22:6, and cLPA 14:0 and the risk of developing HFpEF. However, in patients with diagnosed HFpEF, the levels of LPE 18:0, LPA 16:0, and cLPA 20:4 were reduced. This pattern of shifting associations observed as individuals transition from PreDx-HFpEF to PostDx-HFpEF extends beyond HDL and oxylipins. Regarding LPIs, the AS160 deficiency increases their levels, which further impairs cardiac contractility.^37^ Our data revealed that three LPI species (LPI 16:1, LPI 18:1, and LPI 18:2) exhibited a positive association with higher chances of diagnosed HFpEF. The role of LPGs in cardiovascular system was less evident. Generally, signalling lipids belonging to the same subclass exhibited consistent trends across different analyses.

Our study has two key strengths. First, the targeted lipidomic approach facilitates systematic and reliable investigation of potentially relevant signalling lipids, enabling in-depth exploration of lipid perturbations in HFpEF. Second, we collected samples of both pre- and post-diagnosis, which offers the valuable opportunity to characterise the lipidomic shifts from the pre-clinical to the established disease stage. However, this study is not without limitations. The sample size was relatively small and imbalanced, requiring further validation of the findings in larger cohorts. In particular, the limited size of the PostDx-HFpEF subgroup may reduce statistical power and increase susceptibility to random variation, emphasising the exploratory nature of post-diagnosis findings. Furthermore, relevant clinical biomarkers including pro-BNP and additional lipid indicators such as LDL and HDL particle characteristics were not collected and evaluated, which limits the comprehensive contextualisation of the lipidomic results.

The generalisability of these findings may be limited by the use of a single, community-based European cohort and the relatively small number of post-diagnosis HFpEF cases; therefore, validation in more diverse populations and larger cohorts is warranted.

In conclusion, this study demonstrates that HFpEF is accompanied by dynamic alterations in signalling lipids rather than uniform changes in the overall lipid profile. Subtle elevations in specific non-esterified oxylipins (derived from DHA, AA, EPA) and lysophospholipids were observed prior to diagnosis and showed predictive associations with future HFpEF onset, whereas these lipid signatures were attenuated after clinical diagnosis, alongside changes in HDL. Together, these findings highlight signalling lipids as sensitive indicators of disease stage and progression, providing a framework for future studies on early disease characterisation and biomarker development in HFpEF.

## Contributors

MB and TH contributed to the conception and design of the study, as well as funding acquisition. SB and RG were responsible for the acquisition of clinical data. LZ and TT contributed to clinical data curation, with LZ additionally involved in signalling lipid data acquisition. LZ and AK participated in the analysis and interpretation of data. LZ drafted the original manuscript. AH, LL and TH provided supervision. The author LZ and AH verified the data and had full access to all the raw data in the study. All authors contributed to the revision of the manuscript. All authors read and approved the final version of the manuscript.

## Data sharing statement

The clinical variable datasets used and analysed in the current study are available from the corresponding author upon reasonable request. The metabolomics variable datasets used and analysed in the current study are available in the MetaboLights repository under accession number MTBLS12766.

## AI usage statement

During the preparation of this work, the authors used Grammarly to assist with grammar and spelling corrections. The authors have reviewed and confirmed the validity of the text and take full responsibility for the content of the publication.

## Declaration of interests

The authors declare that they have no competing interests.
