# Supplementary material for: Pre- and post-diagnosis signalling lipid profiles in heart failure with preserved ejection fraction: a prospective cohort study
Source: eBioMedicine. 2026 Mar 27;126:106235. doi: 10.1016/j.ebiom.2026.106235 (PMC13059115; doi:10.1016/j.ebiom.2026.106235)
Supplement: Supplementary Materials [file mmc1.pdf]

Supplementary Material

Index of contained information:

- Figure S1. Prevalence of hypertension, hyperlipidaemia, diabetes, CKD stages in Control, PreDx-HFpEF and PostDx-HFpEF group
- Figure S2. Proportion of significantly correlated metabolites by different clinical variables
- Figure S3. Hazard ratios of relevant clinical variables by Cox models (Control vs PreDx-HFpEF)
- Figure S4. Violin plots of cholesterol and HDL before lipid-lowering drug adjustment
- Figure S5. Paired box plots of cholesterol, HDL and significant metabolites
- Figure S6. Violin plots and paired box plots of DHA, AA and EPA.
- Figure S7. Metabolic pathways and functions in cardiovascular system of significant metabolites
- Table S1. Target list of detectable metabolites
- Table S2. Sums and ratios of metabolites
- Table S3. Summary of missing values and rates for clinical variables.
- Protocol S1. Steroid measurement

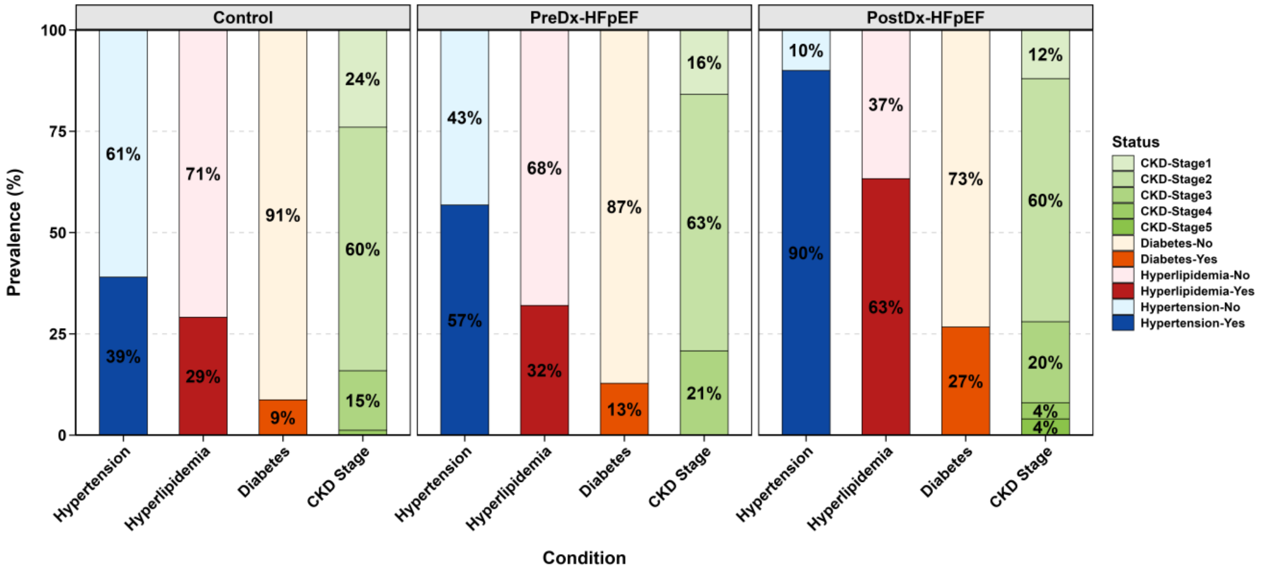

Figure S1. Prevalence of hypertension, hyperlipidaemia, diabetes, and CKD stages in the Control, PreDx-HFpEF, and PostDx-HFpEF groups.

The figure shows the proportion of participants with each clinical condition in the three study groups. Prevalence was calculated as the percentage of individuals meeting the predefined clinical criteria within each group. This analysis is descriptive in nature, and no formal statistical comparisons between groups were performed. Data were obtained from 172 control, 125 PreDx-HFpEF, and 30 PostDx-HFpEF samples.

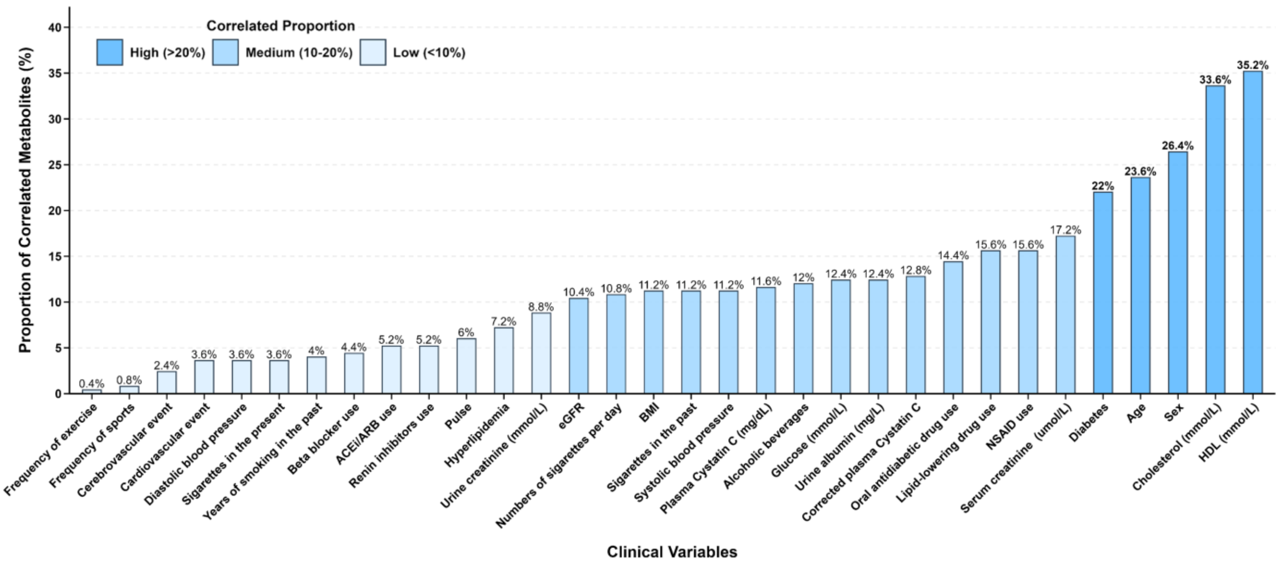

Figure S2. Proportion of significantly correlated metabolites by different clinical variables.

Spearman correlation analysis was used to evaluate the statistical significance of correlations between metabolites and various clinical variables. The threshold for defining statistical significance was set at an adjusted p-value (p.adj) < 0.05. The p.adj values were derived from Spearman correlation analyses with FDR correction. Data were obtained from 172 control, 125 PreDx-HFpEF, and 30 PostDx-HFpEF samples.

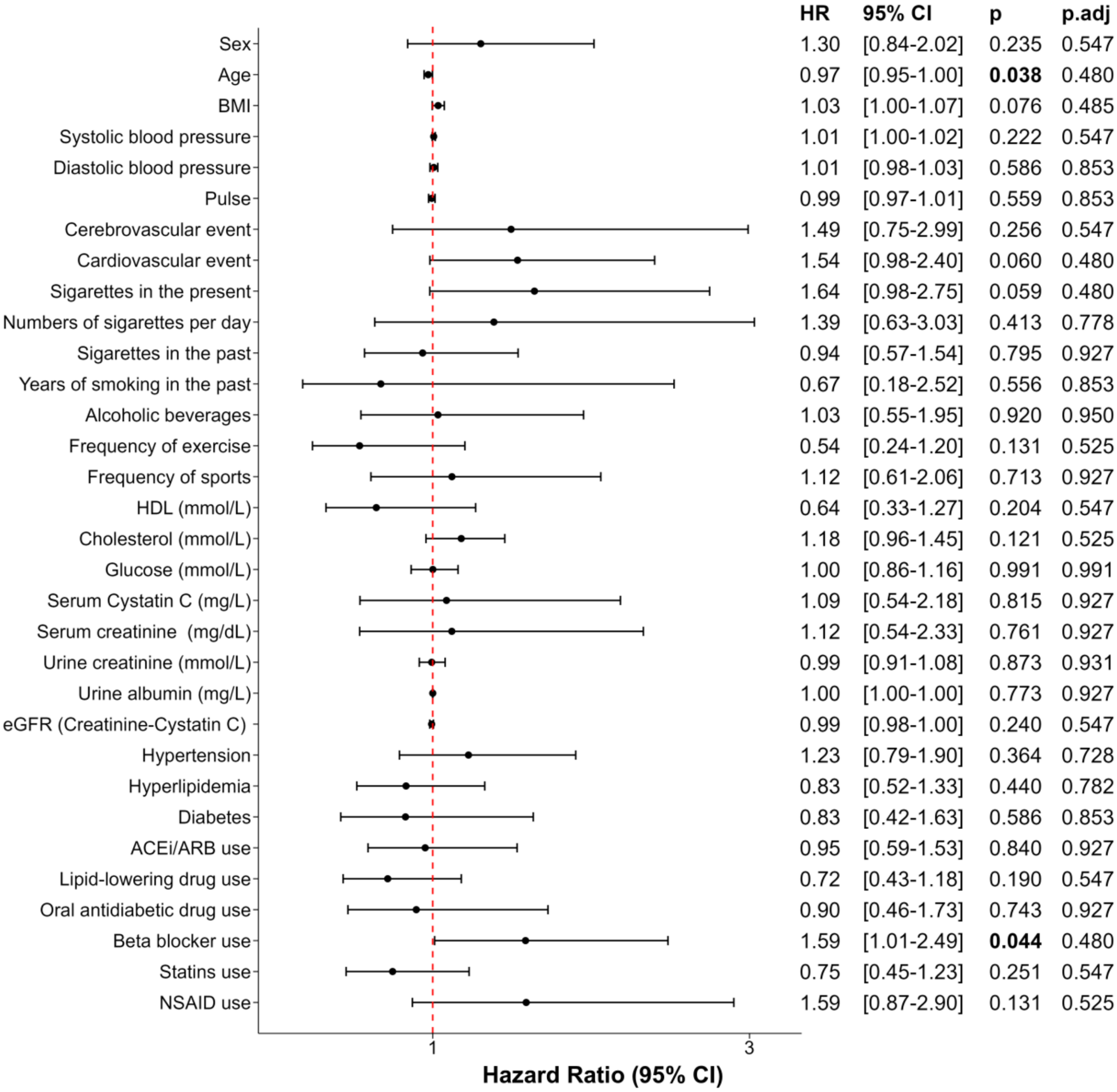

	HR	95% CI	p	p.adj
Sex	1.30	[0.84-2.02]	0.235	0.547
Age	0.97	[0.95-1.00]	**0.038**	0.480
BMI	1.03	[1.00-1.07]	0.076	0.485
Systolic blood pressure	1.01	[1.00-1.02]	0.222	0.547
Diastolic blood pressure	1.01	[0.98-1.03]	0.586	0.853
Pulse	0.99	[0.97-1.01]	0.559	0.853
Cerebrovascular event	1.49	[0.75-2.99]	0.256	0.547
Cardiovascular event	1.54	[0.98-2.40]	0.060	0.480
Sigarettes in the present	1.64	[0.98-2.75]	0.059	0.480
Numbers of sigarettes per day	1.39	[0.63-3.03]	0.413	0.778
Sigarettes in the past	0.94	[0.57-1.54]	0.795	0.927
Years of smoking in the past	0.67	[0.18-2.52]	0.556	0.853
Alcoholic beverages	1.03	[0.55-1.95]	0.920	0.950
Frequency of exercise	0.54	[0.24-1.20]	0.131	0.525
Frequency of sports	1.12	[0.61-2.06]	0.713	0.927
HDL (mmol/L)	0.64	[0.33-1.27]	0.204	0.547
Cholesterol (mmol/L)	1.18	[0.96-1.45]	0.121	0.525
Glucose (mmol/L)	1.00	[0.86-1.16]	0.991	0.991
Serum Cystatin C (mg/L)	1.09	[0.54-2.18]	0.815	0.927
Serum creatinine (mg/dL)	1.12	[0.54-2.33]	0.761	0.927
Urine creatinine (mmol/L)	0.99	[0.91-1.08]	0.873	0.931
Urine albumin (mg/L)	1.00	[1.00-1.00]	0.773	0.927
eGFR (Creatinine-Cystatin C)	0.99	[0.98-1.00]	0.240	0.547
Hypertension	1.23	[0.79-1.90]	0.364	0.728
Hyperlipidemia	0.83	[0.52-1.33]	0.440	0.782
Diabetes	0.83	[0.42-1.63]	0.586	0.853
ACEi/ARB use	0.95	[0.59-1.53]	0.840	0.927
Lipid-lowering drug use	0.72	[0.43-1.18]	0.190	0.547
Oral antidiabetic drug use	0.90	[0.46-1.73]	0.743	0.927
Beta blocker use	1.59	[1.01-2.49]	**0.044**	0.480
Statins use	0.75	[0.45-1.23]	0.251	0.547
NSAID use	1.59	[0.87-2.90]	0.131	0.525

Figure S3. Hazard ratios of relevant clinical variables by Cox models (Control vs PreDx-HFpEF).

Hazard ratios represent the risk of developing HFpEF per 1-SD increase in the relative abundance of lipid targets. Incident HFpEF was defined as the outcome event, and time-to-event was calculated from baseline assessment to HFpEF diagnosis or end of follow-up. The p and p.adj values were derived from Cox proportional-hazards models without or with FDR correction, respectively. Data were obtained from 172 control, 125 PreDx-HFpEF, and 30 PostDx-HFpEF samples.

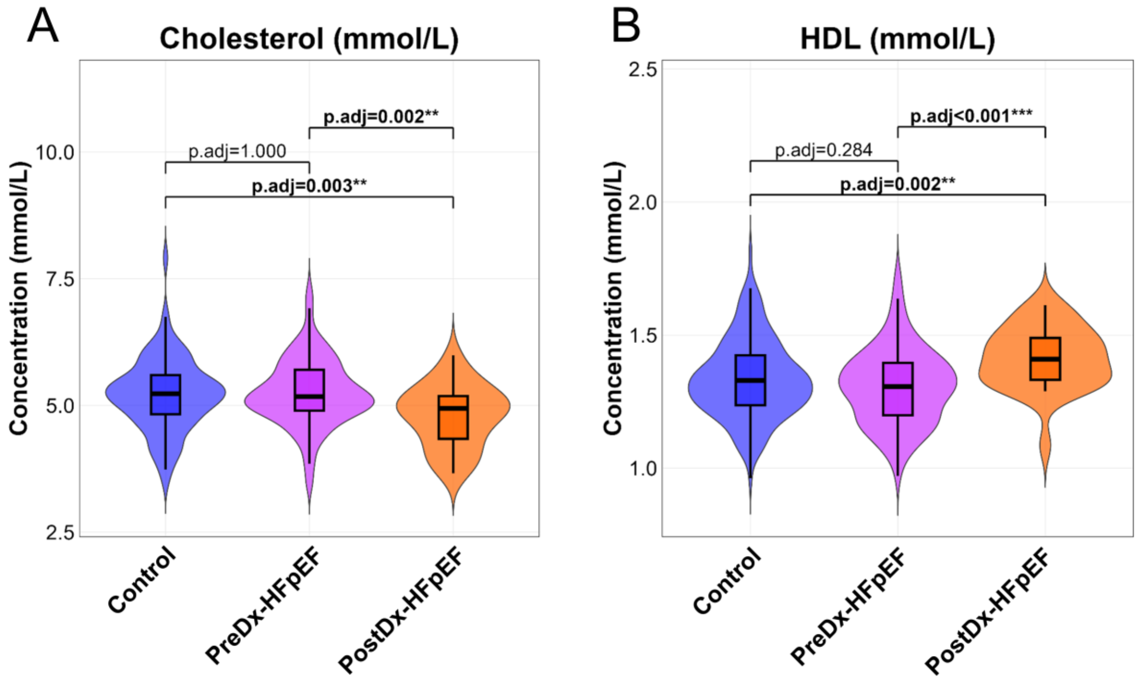

Figure S4. Violin plots of cholesterol and HDL before lipid-lowering drug adjustment.

Violin plots display the distribution of cholesterol and HDL levels across the Control, PreDx-HFpEF, and PostDx-HFpEF groups. Group differences were assessed using Tukey's HSD tests with FDR correction for multiple comparisons. *p.adj < 0.05; **p.adj < 0.01; ***p.adj < 0.001. Data were obtained from 172 control, 125 PreDx-HFpEF, and 30 PostDx-HFpEF samples.

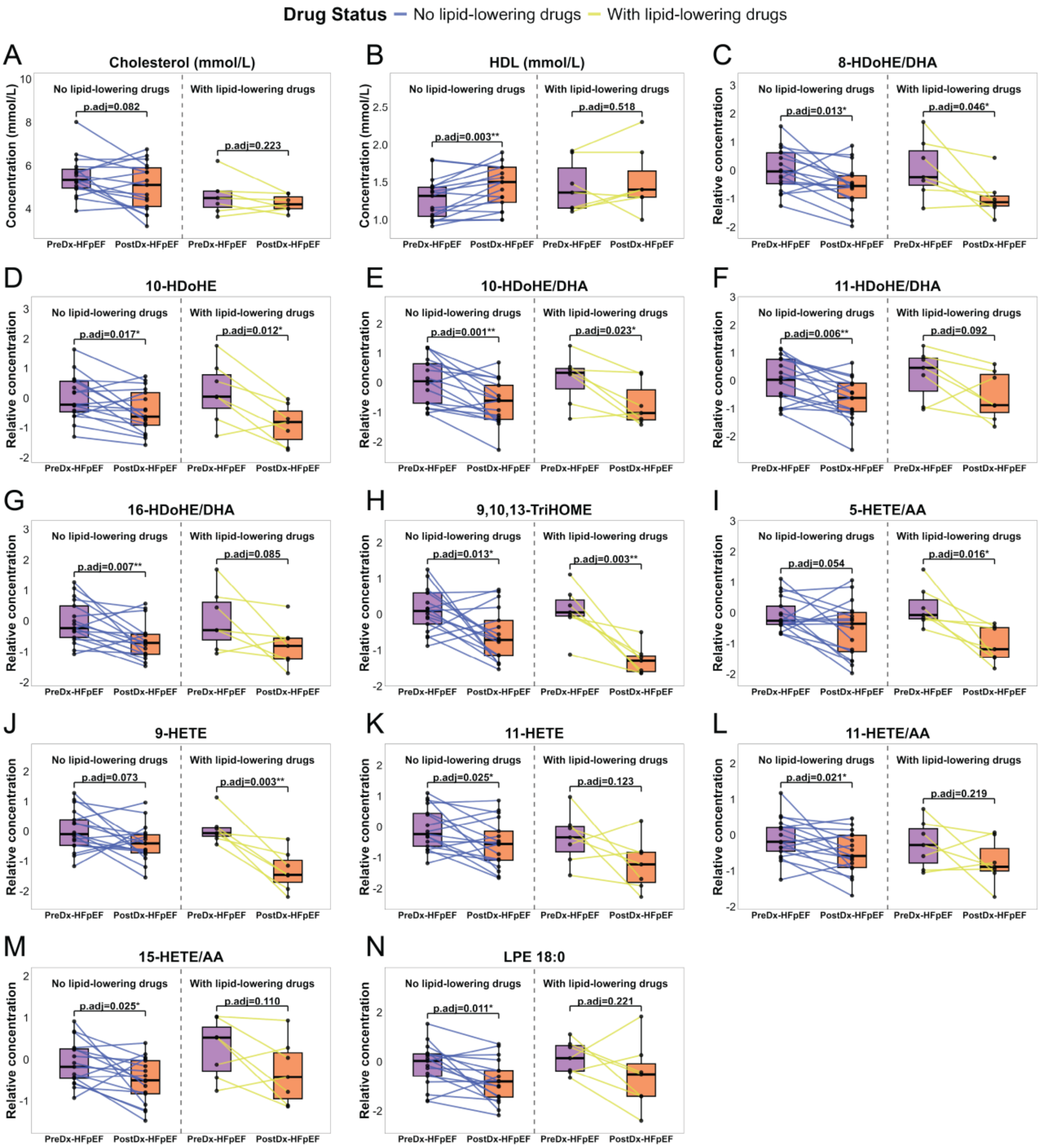

Figure S5. Paired box plots of cholesterol, HDL and significant metabolites.

Participants were stratified according to lipid-lowering drug use (treated vs untreated). Unadjusted cholesterol (A), HDL (B), and all displayed metabolites were shown separately within each stratum. Plotted metabolites were selected based on their identification as significant targets in at least two comparisons, including: (C) 8-HDoHE/DHA, (D) 10-HDoHE, (E) 10-HDoHE/DHA, (F) 11-HDoHE/DHA, (G) 16-HDoHE/DHA, (H) 9,10,13-TriHOME, (I) 5-HETE/AA, (J) 9-HETE, (K) 11-HETE, (L) 11-HETE/AA, (M) 15-HETE/AA, and (N) LPE 18:0. Paired differences between PreDx-HFpEF and PostDx-HFpEF were assessed using paired t tests within each stratum, with FDR correction applied for multiple testing. *p.adj < 0.05; **p.adj < 0.01; ***p.adj < 0.001. Data were obtained from 30 paired PreDx-HFpEF and PostDx-HFpEF samples.

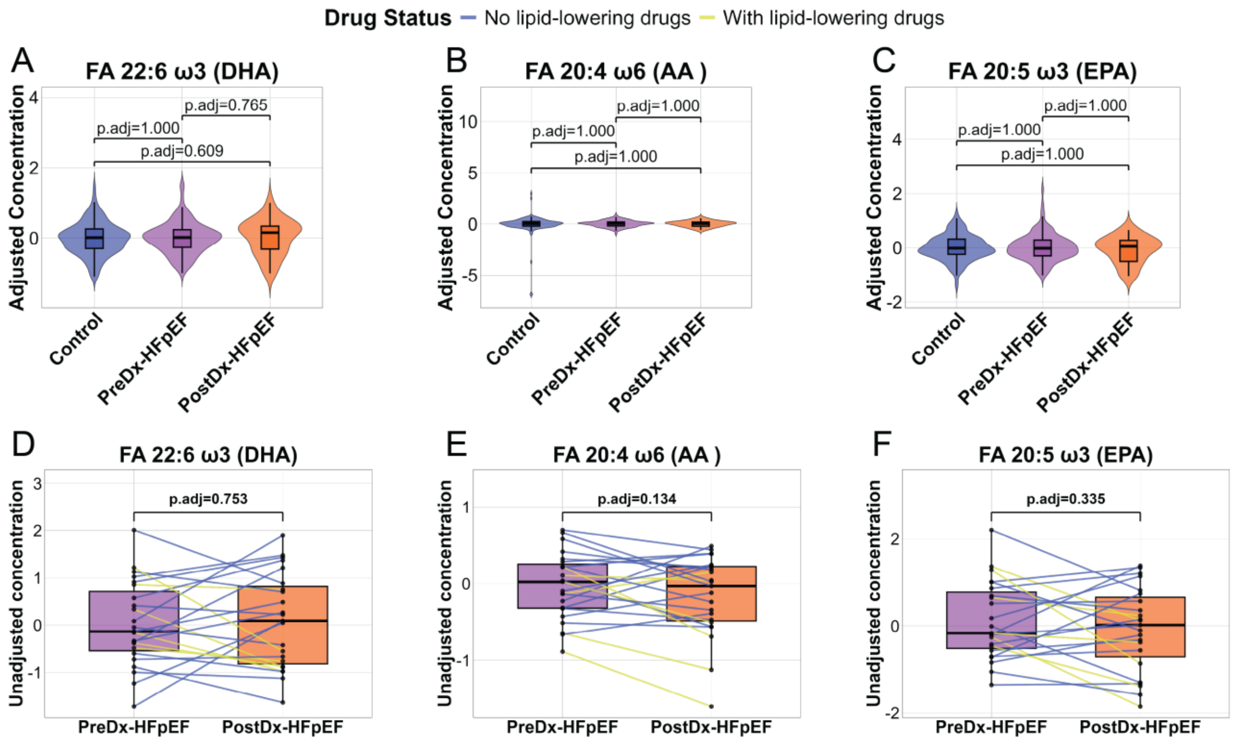

Figure S6. Violin plots and paired box plots of DHA, AA and EPA.

Violin plots depict the distribution of adjusted DHA, AA, and EPA levels across the Control, PreDx-HFpEF, and PostDx-HFpEF groups for (A) DHA, (B) AA, and (C) EPA. Adjustments were performed for age, sex, diabetes status, and the use of lipid-lowering drugs, NSAIDs, and antidiabetic drugs. Group differences in panels A to C were assessed using Tukey's HSD tests, with FDR correction applied for multiple comparisons. Paired box plots depict paired samples from the PreDx-HFpEF and PostDx-HFpEF groups for (D) DHA, (E) AA, and (F) EPA using unadjusted values. Paired differences in panels D to F were assessed using paired t tests with FDR correction. Data were obtained from 172 control, 125 PreDx-HFpEF, and 30 PostDx-HFpEF samples.

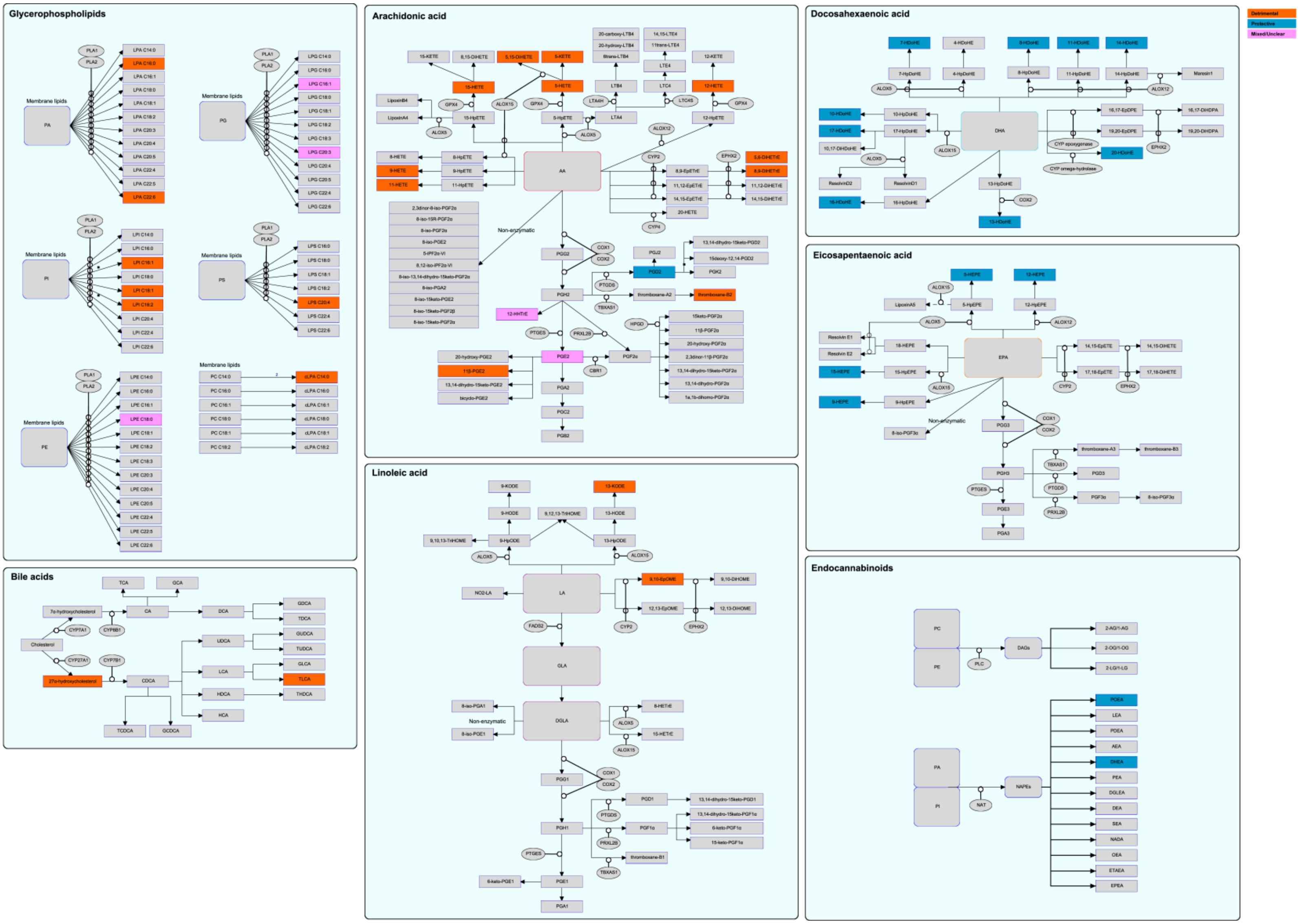

Figure S7. Chemical metabolic pathways of significant metabolites and their reported cardiovascular effects.

Significant metabolites identified in the present study (adjusted p-value < 0.05) were mapped to chemical metabolic pathways using the KEGG and HMDB databases. The figure provides a schematic overview of the major chemical reactions and metabolic routes involving these metabolites. Colours indicate the general direction of reported cardiovascular effects based on existing literature: orange-yellow denotes metabolites predominantly associated with detrimental effects, dark blue denotes metabolites predominantly associated with protective effects, and pink-purple denotes metabolites with mixed or unclear cardiovascular effects. These annotations represent qualitative summaries of current knowledge and do not constitute systematic biological or functional analyses.

Table S1. Target list of detectable metabolites

(For metabolites with multiple IDs, the different IDs represent variations in acyl chain locations and/or double bond locations in the acyl chain. Peaks corresponding to these multiple IDs of the same metabolite were combined during analysis.)

**Metabolites**	**Common name**	**HMDB ID**	**Lipidmaps ID**
FA 16:0 (PA)	Palmitic acid	HMDB0000220	LMFA01010001
FA 18:0 (SA)	Stearic acid	HMDB0000827	LMFA01010018
FA 18:1 ω9 (OA)	Oleic acid	HMDB0000207	LMFA01030002
FA 18:2 ω6 (LA)	Linoleic acid	HMDB0000673	LMFA01030120
FA 18:3 ω3 (ALA)	Alpha-Linolenic acid (alpha-LA)	HMDB0001388	LMFA01030152
FA 18:3 ω6 (GLA)	Gamma-Linolenic acid (gamma-LA)	HMDB0003073	LMFA01030141
FA 20:3 ω3 (DALA)	Dihomo-alpha-linolenic acid (DALA)	HMDB0060039	LMFA01030159
FA 20:3 ω6 (DGLA)	Dihomo-gamma-linolenic acid (DGLA)	HMDB0002925	LMFA01030158
FA 20:3 ω9 (MA)	5,8,11-Eicosatrienoic acid (Mead acid)	HMDB0010378	LMFA01030381
FA 20:4 ω6 (AA)	Arachidonic acid (AA)	HMDB0001043	LMFA01030001
FA 20:5 ω3 (EPA)	Eicosapentaenoic acid (EPA)	HMDB0001999	LMFA01030759
FA 22:4 ω6 (ADA)	Adrenic acid	HMDB0002226	LMFA01030178
FA 22:5 ω3 (ω3-DPA)	Docosapentaenoic acid (omega3-DPA)	HMDB0006528	LMFA04000044
FA 22:5 ω6 (ω6-DPA)	Docosapentaenoic acid (omega6-DPA)	HMDB0001976	LMFA04000064
FA 22:6 ω3 (DHA)	Docosahexaenoic acid (DHA)	HMDB0002183	LMFA01030185
5-HETE	5-HETE	HMDB0011134	LMFA03060084
9-HETE	9-HETE	HMDB10222	LMFA03060089
11-HETE	11-HETE	HMDB04682	LMFA03060085
12-HETE	12-HETE	HMDB06111	LMFA03060088
15-HETE	15-HETE	HMDB0003876	LMFA03060087
20-HETE	20-HETE	HMDB0005998	LMFA03060009
5-KETE	5-Oxo-ETE	HMDB0010217	LMFA03060011
5,6-DiHETrE	(+/-)5,6-DiHETrE	HMDB0002343	LMFA03050004
8,9-DiHETrE	(+/-)8,9-DiHETrE	HMDB0002311	LMFA03050006
11,12-DiHETrE	(+/-)11,12-DiHETrE	HMDB0002314	LMFA03050008
14,15-DiHETrE	(+/-)14,15-DiHETrE	HMDB0002265	LMFA03050010
12-HHTrE	12S-HHTrE;12-HHT	HMDB0012535	LMFA03050002
11,12-EpETrE	(+/-)11,12-EpETrE	HMDB10409	LMFA03080004
14,15-EpETrE	(+/-)14,15-EpETrE	HMDB0004264	LMFA03080005
5,15-DiHETE	5,15-DiHETE	HMDB0010216	LMFA03060107
8-HETrE	8(S)-HETrE	HMDB0060052	LMFA03050011
15-HETrE	15S-HETrE	HMDB0005045	LMFA03050007
5-HEPE	(+/-)-5-HEPE	HMDB0005081	LMFA03070027
9-HEPE	(+/-)-9-HEPE	HMDB0060053	LMFA03070029
12-HEPE	(+/-)-12-HEPE	HMDB10202	LMFA03070031
15-HEPE	(+/-)-15-HEPE	HMDB0010209	LMFA03070032
18-HEPE	(+/-)-18-HEPE	HMDB0012611	LMFA03070033

**Metabolites**	**Common name**	**HMDB ID**	**Lipidmaps ID**
14,15-DiHETE	14,15-DiHETE	HMDB0010204	LMFA03060077
17,18-DiHETE	17,18-DiHETE	HMDB0010211	LMFA03060078
7-HDoHE	(+/-)-7-HDoHE	HMDB0060050	LMFA04000025
8-HDoHE	(+/-)-8-HDoHE	HMDB0060051	LMFA04000026
10-HDoHE	(+/-)-10-HDoHE	HMDB0060037	LMFA04000027
11-HDoHE	(+/-)-11-HDoHE	HMDB0060040	LMFA04000028
13-HDoHE	(+/-)-13-HDoHE	HMDB0060043	LMFA04000029
14-HDoHE	(+/-)-14-HDoHE	HMDB0060044	LMFA04000030
16-HDoHE	(+/-)-16-HDoHE	HMDB0060047	LMFA04000031
17-HDoHE	(+/-)-17-HDoHE	HMDB0010213	LMFA04000032
20-HDoHE	(+/-)-20-HDoHE	HMDB0060048	LMFA04000033
10,17-DiHDoHE	Protectin DX	HMDB0244278	LMFA04040003
9-HOTrE	9S-HOTrE; 9-HOTrE	HMDB0031934	LMFA02000024
19,20-DiHDPA	19,20-DiHDPE	HMDB0010214	LMFA04000043
19,20-EpDPE	19(20)-EpDPE	HMDB0013620	LMFA04000038
9,10-EpOME	9(10)-EpOME	HMDB0004701	LMFA02000037
12,13-EpOME	12(13)-EpOME	HMDB0004702	LMFA02000038
9,10-DiHOME	9,10-DiHOME	HMDB0004704	LMFA02000229
12,13-DiHOME	12,13-DiHOME	HMDB0004705	LMFA02000230
9,10,13-TriHOME	9(S),10(S),13(S)-TriHOME	HMDB0004710	LMFA02000168
9-HODE	9-HODE	HMDB0062652	LMFA02000151
13-HODE	Coriolic acid	HMDB0112194	LMFA02000154
9-KODE	9-OxoODE	HMDB0004669	LMFA02000274
13-KODE	13-Oxo-ODE	HMDB0004668	LMFA02000016
12,13-DiHODE	alpha-12,13-DiHODE	HMDB0010201	LMFA02000046
8-iso-PGA1	8-iso-Prostaglandin A1	HMDB0002236	LMFA03110008
PGA2	Prostaglandin A2	HMDB0002752	LMFA03010035
PGD2	Prostaglandin D2	HMDB0001403	LMFA03010004
PGE2	Prostaglandin E2	HMDB0001220	LMFA03010003
11β-PGE2	11beta-PGE2	HMDB0060041	LMFA03010060
11β-PGF2α	11beta-PGF2alpha	HMDB0010199	LMFA03010036
5-iPF2α-VI	(+/-) 5-iPF2alpha-VI	HMDB0341543	LMFA03110011
8,12-iso-iPF2α-VI	8,12-iso-iPF2alpha-VI		
8-iso-PGF3α	8-iso-PGF3alpha	HMDB0002132	LMFA03110007
20-carboxy-LTB4	20-carboxy-LTB4	HMDB0006059	LMFA03020016
6trans-LTB4	6-trans-Leukotriene B4	HMDB0001085	LMFA03020013
thromboxane-B2	Thromboxane B2	HMDB0003252	LMFA03030002
thromboxane-B3	Thromboxane B3	HMDB0005099	LMFA03030006
PAF C18:2	PC(O-18:2(9Z,12Z)/2:0)		LMGP01020158
1-AG & 2-AG	1-Arachidonoyl Glycerol; 2-Arachidonoyl Glycerol	HMDB0011578; HMDB0004666	LMGL01010032; LMGL01010023
1-LG & 2-LG	1-Linoleoyl Glycerol (18:2); 2-Linoleoyl Glycerol (18:2)	HMDB0011568; HMDB0011538	LMGL01010006; LMGL01010033

Metabolites	Common name	HMDB ID	Lipidmaps ID
1-OG & 2-OG	1-Oleoyl Glycerol (18:1); 2-Oleoyl Glycerol (18:1)	HMDB0094684; HMDB0011537	LMGL01010005; LMGL01010024
AEA	Anandamide;Anandamide (20:4, n-6)	HMDB0004080	LMFA08040001
POEA	Palmitoleoyl Ethanolamide	HMDB0013648	LMFA08040043
DHEA	Docosahexaenoyl Ethanolamide;Anandamide (22:6, n-3)	HMDB0013658	LMFA08040009
10-NO2-OA	10-nitro-9E-octadecenoic acid	HMDB0062737	LMFA01120003
NO2-αLA	10-Nitrolinoleic acid; 10-Nitrolinoleate	HMDB0005049	LMFA01120001
LPA 14:0	Lysophosphatidic acid (14:0)	HMDB0062321	LMGP10050007
LPA 16:0	Lysophosphatidic acid (16:0)	HMDB07853; HMDB07849	LMGP10050006; LMGP10050042
LPA 16:1	Lysophosphatidic acid (16:1)	HMDB0062323	LMGP10050016
LPA 18:0	Lysophosphatidic acid (18:0)	HMDB07854; HMDB07850	LMGP10050005; LMGP10050043
LPA 18:1	Lysophosphatidic acid (18:1)	HMDB07855; HMDB07851	LMGP10050008; LMGP10050014
LPA 18:2	Lysophosphatidic acid (18:2)	HMDB0007856; HMDB07852	LMGP10050017; LMGP10050044
LPA 20:3	Lysophosphatidic acid (20:3)	HMDB0062313	LMGP10050028
LPA 20:4	Lysophosphatidic acid (20:4)	HMDB0062312	LMGP10050013
LPA 20:5	Lysophosphatidic acid (20:5)	HMDB0062308	LMGP10050033
LPA 22:4	Lysophosphatidic acid (22:4)	HMDB0062310	LMGP10050020
LPA 22:5	Lysophosphatidic acid (22:5)	HMDB0114753; HMDB0114754	
LPA 22:6	Lysophosphatidic acid (22:6)	HMDB0114755	LMGP10050019
cLPA 14:0	Cyclic-Lysophosphatidic acid (14:0)		LMGP00000070
cLPA 16:1	Cyclic-Lysophosphatidic acid (16:1)		
cLPA 18:2	Cyclic-Lysophosphatidic acid (18:2)	HMDB0007007	
cLPA 18:0	Cyclic-Lysophosphatidic acid (18:0)	HMDB0007004	LMGP00000055
cLPA 18:1	Cyclic-Lysophosphatidic acid (18:1)	HMDB0007006	LMGP00000056
cLPA 20:4	Cyclic-Lysophosphatidic acid (20:4)		
LPE 14:0	Lysophosphatidylethanolamine (14:0)	HMDB0011500; HMDB0011470	LMGP02050003; LMGP02050033
LPE 16:0	Lysophosphatidylethanolamine (16:0)	HMDB0011503; HMDB0011473	LMGP02050002; LMGP02050036
LPE 16:1	Lysophosphatidylethanolamine (16:1)	HMDB0011504; HMDB0011474	LMGP02050010; LMGP02050037
LPE 18:0	Lysophosphatidylethanolamine (18:0)	HMDB0011130; HMDB0011129	LMGP02050001; LMGP02050038
LPE 18:1	Lysophosphatidylethanolamine (18:1)	HMDB0011475; HMDB0011476; HMDB0011505; HMDB0011506	LMGP02050039; LMGP02050040; LMGP02050064; LMGP02050004
LPE 18:2	Lysophosphatidylethanolamine (18:2)	HMDB0011507; HMDB0011477	LMGP02050011; LMGP02050041
LPE 18:3	Lysophosphatidylethanolamine (18:3)	HMDB0011478; HMDB0011479; HMDB0011508; HMDB0011509	LMGP02050042; LMGP02050043; LMGP02050017; LMGP02050029
LPE 20:3	Lysophosphatidylethanolamine (20:3)	HMDB0011516; HMDB0011486; HMDB0011514; HMDB0011484; HMDB0011515; HMDB0011485	LMGP02050022; LMGP02050050; LMGP02050065; LMGP02050048; LMGP02050066; LMGP02050049
LPE 20:4	Lysophosphatidylethanolamine (20:4)	HMDB0011517; HMDB0011487; HMDB0011518; HMDB0011488	LMGP02050009; LMGP02050051; LMGP02050067; LMGP02050052
LPE 20:5	Lysophosphatidylethanolamine (20:5)	HMDB0011519; HMDB0011489	LMGP02050027; LMGP02050053
LPE 22:4	Lysophosphatidylethanolamine (22:4)	HMDB0011523; HMDB0011493	LMGP02050014; LMGP02050057
LPE 22:5	Lysophosphatidylethanolamine (22:5)	HMDB0011494; HMDB0011495; HMDB0011524; HMDB0011525	LMGP02050058; LMGP02050059; LMGP02050069; LMGP02050070
LPE 22:6	Lysophosphatidylethanolamine (22:6)	HMDB0011526; HMDB0011496	LMGP02050013; LMGP02050060
LPG 14:0	Lysophosphatidylglycerol (14:0)		LMGP04050012

Metabolites	Common name	HMDB ID	Lipidmaps ID
LPG 16:0	Lysophosphatidylglycerol (16:0)	HMDB0240601	LMGP04050008
LPG 16:1	Lysophosphatidylglycerol (16:1)		LMGP04050013
LPG 18:0	Lysophosphatidylglycerol (18:0)		LMGP04050009
LPG 18:1	Lysophosphatidylglycerol (18:1)	HMDB0240602	LMGP04050006
LPG 18:2	Lysophosphatidylglycerol (18:2)	HMDB0240600	LMGP04050014
LPG 18:3	Lysophosphatidylglycerol (18:3)		LMGP04050032
LPG 20:3	Lysophosphatidylglycerol (20:3)		LMGP04050025
LPG 20:4	Lysophosphatidylglycerol (20:4)		LMGP04050010
LPG 22:4	Lysophosphatidylglycerol (22:4)		LMGP04050017
LPG 22:6	Lysophosphatidylglycerol (22:6)		LMGP04050016
LPI 14:0	Lysophosphatidylinositol (14:0)		LMGP06050008
LPI 16:0	Lysophosphatidylinositol (16:0)	HMDB0061695	LMGP06050002
LPI 16:1	Lysophosphatidylinositol (16:1)		LMGP06050009
LPI 18:0	Lysophosphatidylinositol (18:0)	HMDB0240261	LMGP06050004
LPI 18:1	Lysophosphatidylinositol (18:1)	HMDB0061693	LMGP06050005
LPI 18:2	Lysophosphatidylinositol (18:2)	HMDB0240597	LMGP06050010
LPI 20:4	Lysophosphatidylinositol (20:4)	HMDB0062722	LMGP06050006
LPI 22:4	Lysophosphatidylinositol (22:4)		LMGP06050013
LPS 16:0	Lysophosphatidylserine (16:0)	HMDB0240605	LMGP03050002
LPS 18:0	Lysophosphatidylserine (18:0)	HMDB0240606	LMGP03050006
LPS 18:1	Lysophosphatidylserine (18:1)	HMDB0240603	LMGP03050001
LPS 20:4	Lysophosphatidylserine (20:4)		LMGP03050007
LPS 22:4	Lysophosphatidylserine (22:4)		LMGP03050014
LPS 22:6	Lysophosphatidylserine (22:6)		LMGP03050013
Sphinganine18.0	Sphinganine 18:0		
Sphingosine18.1	Sphinganine-1-phosphate (18:0)		
Sphinganine-1-phosphate 18:0	Sphingosine 18:1	HMDB0001383	LMSP01050002
Sphingosine-1-phosphate 16:1	Sphingosine-1-phosphate (16:1)	HMDB0060061	LMSP01050005
Sphingosine-1-phosphate 18:1	Sphingosine-1-phosphate (18:1)	HMDB0000277	LMSP01050001
Sphingosine-1-phosphate 18:2	Sphingosine-1-phosphate (18:2)		
CA	Cholic acid	HMDB0000619	LMST04010001
CDCA	Chenodeoxycholic acid	HMDB0000518	LMST04010032
DCA	Deoxycholic acid	HMDB0000626	LMST04010040
GCA	Glycocholic acid	HMDB00138	LMST05030001
GCDCA	Glycochenodeoxycholic acid	HMDB0000637	LMST05030008
GDCA	Glycodeoxycholic acid	HMDB0000631	LMST05030006
GLCA	Glycolithocholic Acid	HMDB0000698	LMST05030009
GUDCA	Glycoursodeoxycholic acid	HMDB0000708	LMST05030016
HCA	Hyocholic acid	HMDB0000760	LMST04010064
HDCA	Hyodeoxycholic acid	HMDB0000733	LMST04010024
LCA	Lithocholic acid	HMDB0000761	LMST04010003

**Metabolites**	**Common name**	**HMDB ID**	**Lipidmaps ID**
TCA	Taurocholic acid	HMDB0000036	LMST05040001
TCDCA	Taurochenodesoxycholic acid	HMDB0000951	LMST05040005
TDCA	Taurodeoxycholic acid	HMDB0000896	LMST05040013
THDCA	Taurohyodeoxycholic Acid		
TLCA	Taurolithocholic acid	HMDB00722	LMST05040003
TLCA-3S	Taurolithocholic acid 3-sulfate	HMDB0002580	LMST05020003
TUDCA	Tauroursodeoxycholic acid	HMDB0000874	LMST05040015
UDCA	Ursodeoxycholic acid	HMDB0000946	LMST04010033
11-Deoxycorticosterone	11-Deoxycorticosterone	HMDB0000016	LMST02030087
11-Deoxycortisol	11-Deoxycortisol	HMDB0000015	LMST02030086
17α-Hydroxyprogesterone	17α-Hydroxyprogesterone	HMDB0000374	LMST02030161
21-Deoxycortisol	21-Deoxycortisol	HMDB0004030	LMST02030195
24-Hydroxycholesterol	24-Hydroxycholesterol	HMDB0001419	LMST01010164
27-Hydroxycholesterol	27-Hydroxycholesterol	HMDB0002103	LMST01010057
Androstenedione	Androstenedione	HMDB0000053	LMST02020007
Corticosterone	Corticosterone	HMDB0001547	LMST02030186
Cortisol	Cortisol	HMDB0000063	LMST02030001
Cortisone	Cortisone	HMDB0002802	LMST02030090
Dehydroepiandrosterone	Dehydroepiandrosterone	HMDB0000077	LMST02020021
Testosterone	Testosterone	HMDB0000234	LMST02020002
22(R)-Hydroxycholesterol	22(R)-Hydroxycholesterol	HMDB0004035	LMST01010086
25-Hydroxycholesterol	25-Hydroxycholesterol	HMDB0006247	LMST01010018
Estrone glucuronide	Estrone glucuronide	HMDB0004483	LMST05010011
Estrone sulfate	Estrone sulfate	HMDB0001425	LMST02010043
Progesterone	Progesterone	HMDB0001830	LMST02030159

Table S2. Sums and ratios of metabolites

Sums of metabolites	Lipid subclass	Metabolites
ω-3 fatty acids	Fatty acid	FA 18:3 ω3 (ALA)<br>FA 20:3 ω3 (DALA)<br>FA 20:5 ω3 (EPA)<br>FA 22:5 ω3 (ω3-DPA)<br>FA 22:6 ω3 (DHA)
ω-6 fatty acids	Fatty acid	FA 18:2 ω6 (LA)<br>FA 18:3 ω6 (GLA)<br>FA 20:3 ω6 (DGLA)<br>FA 20:4 ω6 (AA)<br>FA 22:4 ω6 (ADA)<br>FA 22:5 ω6 (ω6-DPA)

Precursor metabolites	Metabolites	Metabolite ratios
ω3-FA	ω6-FA	ω6-FA/ω3-FA
FA 20:4 ω6 (AA)	5-HETE	5-HETE/AA
FA 20:4 ω6 (AA)	9-HETE	9-HETE/AA
FA 20:4 ω6 (AA)	11-HETE	11-HETE/AA
FA 20:4 ω6 (AA)	12-HETE	12-HETE/AA
FA 20:4 ω6 (AA)	15-HETE	15-HETE/AA
FA 20:4 ω6 (AA)	20-HETE	20-HETE/AA
FA 20:4 ω6 (AA)	5-KETE	5-KETE/AA
FA 20:4 ω6 (AA)	5,6-DiHETrE	5,6-DiHETrE/AA
FA 20:4 ω6 (AA)	8,9-DiHETrE	8,9-DiHETrE/AA
FA 20:4 ω6 (AA)	11,12-DiHETrE	11,12-DiHETrE/AA
FA 20:4 ω6 (AA)	14,15-DiHETrE	14,15-DiHETrE/AA
FA 20:4 ω6 (AA)	12-HHTrE	12-HHTrE/AA
FA 20:4 ω6 (AA)	11,12-EpETrE	11,12-EpETrE/AA
FA 20:4 ω6 (AA)	14,15-EpETrE	14,15-EpETrE/AA
FA 20:4 ω6 (AA)	5,15-DiHETE	5,15-DiHETE/AA
FA 20:5 ω3 (EPA)	5-HEPE	5-HEPE/EPA
FA 20:5 ω3 (EPA)	9-HEPE	9-HEPE/EPA
FA 20:5 ω3 (EPA)	12-HEPE	12-HEPE/EPA
FA 20:5 ω3 (EPA)	15-HEPE	15-HEPE/EPA
FA 20:5 ω3 (EPA)	18-HEPE	18-HEPE/EPA
FA 20:5 ω3 (EPA)	14,15-DiHETE	14,15-DiHETE/EPA
FA 20:5 ω3 (EPA)	17,18-DiHETE	17,18-DiHETE/EPA
FA 22:6 ω3 (DHA)	7-HDoHE	7-HDoHE/DHA
FA 22:6 ω3 (DHA)	8-HDoHE	8-HDoHE/DHA
FA 22:6 ω3 (DHA)	10-HDoHE	10-HDoHE/DHA
FA 22:6 ω3 (DHA)	11-HDoHE	11-HDoHE/DHA
FA 22:6 ω3 (DHA)	13-HDoHE	13-HDoHE/DHA
FA 22:6 ω3 (DHA)	14-HDoHE	14-HDoHE/DHA
FA 22:6 ω3 (DHA)	16-HDoHE	16-HDoHE/DHA
FA 22:6 ω3 (DHA)	17-HDoHE	17-HDoHE/DHA
FA 22:6 ω3 (DHA)	20-HDoHE	20-HDoHE/DHA
FA 18:2 ω6 (LA)	9.10-EpOME	9.10-EpOME/LA
FA 18:2 ω6 (LA)	12,13-EpOME	12,13-EpOME/LA
FA 18:2 ω6 (LA)	9,10-DiHOME	9,10-DiHOME/LA
FA 18:2 ω6 (LA)	12,13-DiHOME	12,13-DiHOME/LA
FA 18:2 ω6 (LA)	9,10,13-TriHOME	9,10,13-TriHOME/LA
FA 18:2 ω6 (LA)	9-HODE	9-HODE/LA
FA 18:2 ω6 (LA)	13-HODE	13-HODE/LA
FA 18:2 ω6 (LA)	9-KODE	9-KODE/LA
FA 18:2 ω6 (LA)	13-KODE	13-KODE/LA
FA 20:4 ω6 (AA)	PGA2	PGA2/AA
FA 20:4 ω6 (AA)	PGD2	PGD2/AA
FA 20:4 ω6 (AA)	PGE2	PGE2/AA
FA 20:4 ω6 (AA)	11β-PGE2	11β-PGE2/AA
FA 20:4 ω6 (AA)	11β-PGF2α	11β-PGF2α/AA
FA 20:4 ω6 (AA)	5-iso-PGF2α-VI	5-iso-PGF2α-VI/AA
FA 20:4 ω6 (AA)	8,12-iso-PGF2α-VI	8,12-iso-PGF2α-VI/AA
FA 20:4 ω6 (AA)	20-carboxy-LTB4	20-carboxy-LTB4/AA
FA 20:4 ω6 (AA)	6trans-LTB4	6trans-LTB4/AA
CA	DCA	DCA/CA

**Precursor metabolites**	**Metabolites**	**Metabolite ratios**
CA	GCA	GCA/CA
CDCA	GCDCA	GCDCA/CDCA
DCA	GDCA	GDCA/DCA
LCA	GLCA	GLCA/LCA
UDCA	GUDCA	GUDCA/UDCA
CDCA	LCA	LCA/CDCA
CA	TCA	TCA/CA
CDCA	TCDCA	TCDCA/CDCA
DCA	TDCA	TDCA/DCA
LCA	TLCA	TLCA/LCA
UDCA	TUDCA	TUDCA/UDCA
CDCA	UDCA	UDCA/CDCA

Table S3. Summary of missing values and rates for clinical variables

Characteristic	Control (n = 172)		PreDx-HFpEF (n = 125)		PostDx-HFpEF (n = 30)	
	Number of missing	Missing rate	Number of missing	Missing rate	Number of missing	Missing rate
Age (years)	0	0%	0	0%	0	0%
BMI (kg/m2)	0	0%	0	0%	0	0%
Systolic blood pressure (mmHg)	1	1%	1	1%	0	0%
Diastolic blood pressure (mmHg)	1	1%	1	1%	0	0%
Pulse (beats per minute)	2	1%	1	1%	0	0%
HDL (mmol/L)	4	2%	1	1%	0	0%
Cholesterol (mmol/L)	2	1%	1	1%	0	0%
Glucose (mmol/L)	4	2%	3	2%	0	0%
eGFR (Creatinine-Cystatin C)	9	5%	5	4%	5	17%
Urinary Albumin-Creatinine Ratio (mg/mmol)	0	0%	0	0%	0	0%
CKD stage (n)	9	5%	5	4%	5	17%
Cerebrovascular event in the past (n)	0	0%	0	0%	0	0%
Peripheral event in the past (n)	0	0%	0	0%	0	0%
Cardiovascular event in the past (n)	0	0%	0	0%	0	0%
Venous thromboembolism in the past (n)	0	0%	0	0%	0	0%
Hypertension (n)	0	0%	0	0%	0	0%
Hyperlipidaemia (n)	0	0%	0	0%	0	0%
Diabetes (n)	0	0%	0	0%	0	0%
ACEi/ARB use (n)	10	6%	9	7%	2	7%
Lipid-lowering drug use (n)	10	6%	9	7%	2	7%
Oral antidiabetic drug use (n)	10	6%	9	7%	2	7%
Beta blocker use (n)	14	8%	4	3%	2	7%
Insulin use (n)	14	8%	5	4%	2	7%
NSAID use (n)	14	8%	5	4%	2	7%
Renin inhibitors use (n)	14	8%	5	4%	2	7%
Statins use (n)	10	6%	5	4%	2	7%

Protocol S1. Steroid measurement

Sample Preparation

A 50 µL plasma aliquot was utilized for protein precipitation. Following thawing on ice, 10 µL internal standards solution (ISTD) and 190 µL of ice-cold acetonitrile (ACN) were added to the samples, which were then mixed for 4 minutes using a bullet blender. All samples underwent centrifugation at 4 °C and 15,800 rcf for 10 minutes. Subsequently, 190 µL of the upper aqueous layer was collected and transferred to a clean 1.5 mL tube, which was then dried in a SpeedVac (Thermo Fisher, USA) for approximately 2 hours. The dried residue was reconstituted by adding 35 µL of ice-cold injection solution (50% ACN/50% Milli-Q water), after which the samples were vortexed and centrifuged again at 4 °C and 15,800 rcf for 10 minutes. The resulting supernatants were transferred to injection vials with inserts for LC-MS/MS analysis.

Chromatography

Analyses were performed using a Shimadzu LC-30AD liquid chromatography system coupled to a SCIEX Triple-Quad 7500 mass spectrometer (USA), with separation achieved on a Waters Acquity BEH C18 column (50 mm × 2.1 mm, 1.7 µm) maintained at 40 °C. The dual-pump LC system employed mobile phase A (water containing 0.1% acetic acid) and mobile phase B (90% ACN/10% methanol with 0.1% acetic acid), with pH values ranging from 3.2 to 3.5. The injection volume was 10 µL. Chromatographic separation was carried out at a flow rate of 0.7 mL/min over a 16-minute gradient. The gradient started with 90% mobile phase A and 10% mobile phase B, then adjusted as follows: mobile phase B increased to 13% at 0.75 minutes, 20% at 3 minutes, 54% at 8 minutes, 80% at 10.5 minutes, and 90% at 11.5 minutes, where it was held for 3 minutes. After this, the gradient returned to the initial conditions within 0.3 minutes and remained at the starting conditions until the 16-minute mark.

Mass Spectrometry

The SCIEX Triple-Quad 7500 mass spectrometer employed an electrospray ionization source with the following parameters: interface temperature of 550 °C, curtain gas at 40 psi, collision-activated dissociation (CAD) gas at 9, spray voltage at 4500 V, target cycle time at 400 ms, and both gas 1 and gas 2 at 60 psi. Multiple reaction monitoring (MRM) in polarity switch scanning mode was used to measure analytes and their respective internal standards (ISTDs). Unit resolution was applied in both positive and negative ionization modes.
